# Interfacial Materials for Organic Solar Cells: Recent Advances and Perspectives

**DOI:** 10.1002/advs.201500362

**Published:** 2016-02-18

**Authors:** Zhigang Yin, Jiajun Wei, Qingdong Zheng

**Affiliations:** ^1^ State Key Laboratory of Structural ChemistryFujian Institute of Research on the Structure of MatterChinese Academy of Sciences155 Yangqiao Road WestFuzhouFujian350002P. R. China; ^2^ University of Chinese Academy of Sciences19 Yuquan RoadBeijing100049P. R. China

**Keywords:** organic solar cells, interface engineering, interlayers, semiconductors, energy conversion

## Abstract

Organic solar cells (OSCs) have shown great promise as low‐cost photovoltaic devices for solar energy conversion over the past decade. Interfacial engineering provides a powerful strategy to enhance efficiency and stability of OSCs. With the rapid advances of interface layer materials and active layer materials, power conversion efficiencies (PCEs) of both single‐junction and tandem OSCs have exceeded a landmark value of 10%. This review summarizes the latest advances in interfacial layers for single‐junction and tandem OSCs. Electron or hole transporting materials, including metal oxides, polymers/small‐molecules, metals and metal salts/complexes, carbon‐based materials, organic‐inorganic hybrids/composites, and other emerging materials, are systemically presented as cathode and anode interface layers for high performance OSCs. Meanwhile, incorporating these electron‐transporting and hole‐transporting layer materials as building blocks, a variety of interconnecting layers for conventional or inverted tandem OSCs are comprehensively discussed, along with their functions to bridge the difference between adjacent subcells. By analyzing the structure–property relationships of various interfacial materials, the important design rules for such materials towards high efficiency and stable OSCs are highlighted. Finally, we present a brief summary as well as some perspectives to help researchers understand the current challenges and opportunities in this emerging area of research.

## 1 Introduction

In order to meet the growing global energy demands with a renewable and sustainable resource, converting sunlight energy directly to electricity using photovoltaic technologies is one of the best solutions. Therefore, extensive efforts have been focused on developing new generation photovoltaic technologies such as dye‐sensitized solar cells (DSSCs), organic photovoltaics (OPVs), quantum dot solar cells, perovskite solar cells, etc.^1^, ^2^, ^3^, ^4^ Among these new photovoltaic devices, OPV cells, also named as organic solar cells (OSCs), are potentially easier and cheaper to manufacture than the current silicon‐based technologies. OSCs can be fabricated with the use of large‐area solution processing, while maintaining the salient features of organic devices such as light weight, flexibility, and tunable transparency, etc.^5^, ^6^, ^7^ So far, state‐of‐the‐art power conversion efficiencies (PCEs) exceeding 10% have been achieved for both single‐junction and tandem OSCs.^8^, ^9^, ^10^, ^11^, ^12^, ^13^ At the same time, theoretical studies also demonstrated that PCEs of tandem OSCs could reach a value of 15–20% by optimizing both active layer material properties and device architectures.^14^, ^15^ All these advances will propel OSCs out of the realm of strictly fundamental researches into the industrial manufacturing.

Since the pioneer works in OPVs,^16^, ^17^, ^18^ rapidly increasing PCEs of OSCs are benefited from the developments of new donor/acceptor materials in the active layer and from the innovations of the device structure and geometry.^19^, ^20^, ^21^, ^22^ For example, high PCEs of 9–10% for single‐junction OSCs are achieved by combining [6,6]‐phenyl‐C71‐butyric acid methyl ester (PC_71_BM) as an acceptor with a low‐bandgap polymer, poly({4,8‐bis[(2‐ethylhexyl)oxy]benzo[1,2‐b:4,5‐b′]dithiophene‐2,6‐diyl}{3‐fluoro‐2‐[(2‐ethylhexyl)carbonyl] thieno[3,4‐b]thiophenediyl}) (PTB7) or its derivative PTB7‐Th (poly[4,8‐bis(5‐(2‐ethylhexyl)thiophen‐2‐yl)benzo[1,2‐*b*:4,5‐*b*′]dithiophene‐*co*‐3‐fluorothieno[3,4‐*b*]thiophene‐2‐carboxylate]) as a donor in the active layer.^23^, ^24^, ^25^, ^26^, ^27^ Recently, Yan and co‐workers reported a PCE of 10.8% from inverted single‐junction cells based on a new polymer:fullerene (PffBT4T‐2OD:PC_71_BM) system by controlling the aggregation and morphology of the active layer.^28^ When two photoactive materials with complementary absorption spectra are used to improve light harvesting of tandem OSCs, PCEs over 11% for both polymer and small molecule solar cells have been achieved.^9^, ^29^, ^30^, ^31^

**Figure 1:**
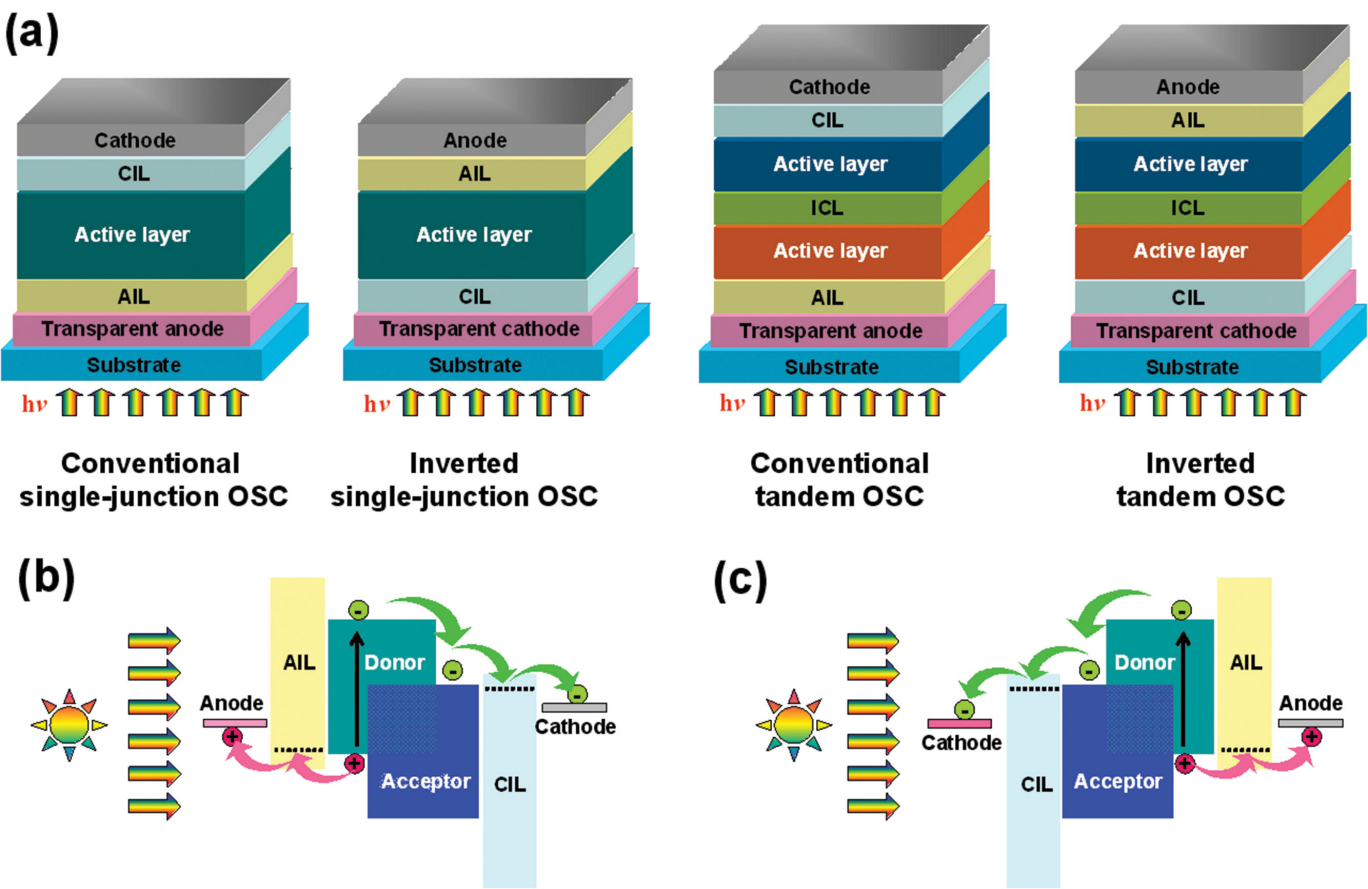
a) Schematic device architectures of conventional and inverted single‐junction/tandem OSCs. **AIL**: anode interface layer; **CIL**: cathode interface layer; **ICL**: interconnecting layer. Schematic illustration of the energy level diagrams and the main charge‐transporting processes in b) conventional and c) inverted OSCs.

Generally, for a typical OSC, a photoactive layer of bulk‐heterojunction (BHJ) or bilayer planar heterojunction is sandwiched between two electrodes (cathode and anode) with their corresponding interlayers. According to the charge flow direction, OSCs can be divided into conventional and inverted devices, as schematically shown in **Figure** **1**a. The initially used architecture is the conventional single‐junction device, which is made of a blended active layer between a modified transparent anode such as indium tin oxide (ITO), and a low work‐function (WF) metal cathode (such as Ca and Al). When the active layer of a conventional OSC is under light irradiation, the photo­generated excitons diffuse towards the donor/acceptor interface and separate into holes and electrons in the highest occupied molecular orbital (HOMO) of the donor, and the lowest unoccupied molecular orbital (LUMO) of the acceptor (Figure 1b), respectively. After that, the separated charge carriers transport within the respective phases of the active layer, until they are collected by the opposite electrodes. Therefore, the energy level structure at electrode interfaces plays an essential role, where an ideal interface needs good Ohmic contact with minimum resistance and high charge selectivity to prevent carriers from reaching the opposite electrodes.^21^ Interfacial materials with adequate WFs to match the energetic levels of donor and acceptor materials are thus desired to insert between the active layer and electrodes, which can enhance the collection efficiencies of holes and electrons on the anode and cathode, respectively. In most of conventional OSCs, poly(3,4‐ethylenedioxythiophene):poly(styrene sulfonate) (PEDOT:PSS) is used as an anode interface layer (AIL) to modify the ITO electrode. However, conventional OSCs usually suffer from rapid degradation and poor lifetime due to the acidic and hydrophilic nature of PEDOT:PSS, and due to the sensitivity of low WF metal anodes to oxygen and moisture.^32^, ^33^ As an alternative, the inverted configuration is a good solution to make PEDOT:PSS‐free devices, where the polarity of charge collection is the opposite of the conventional device (Figure 1c). Inverted OSCs allow the use of less air‐sensitive high WF metals (i.e., Au, and Ag) as the top electrode and a low WF metal oxide cathode interface layer (CIL) modified ITO as the transparent cathode, leading to a better stability compared to conventional OSCs. Besides the AILs and CILs, an interconnecting layer (ICL) is required to connect the subcells in conventional or inverted tandem OSCs (Figure 1a). The ICL is responsible for extracting holes and electrons from the adjacent subcells and then completing the charge recombination within them. Accordingly, the performance of tandem devices greatly depends on the choices and properties of ICL materials.

**Figure 2:**
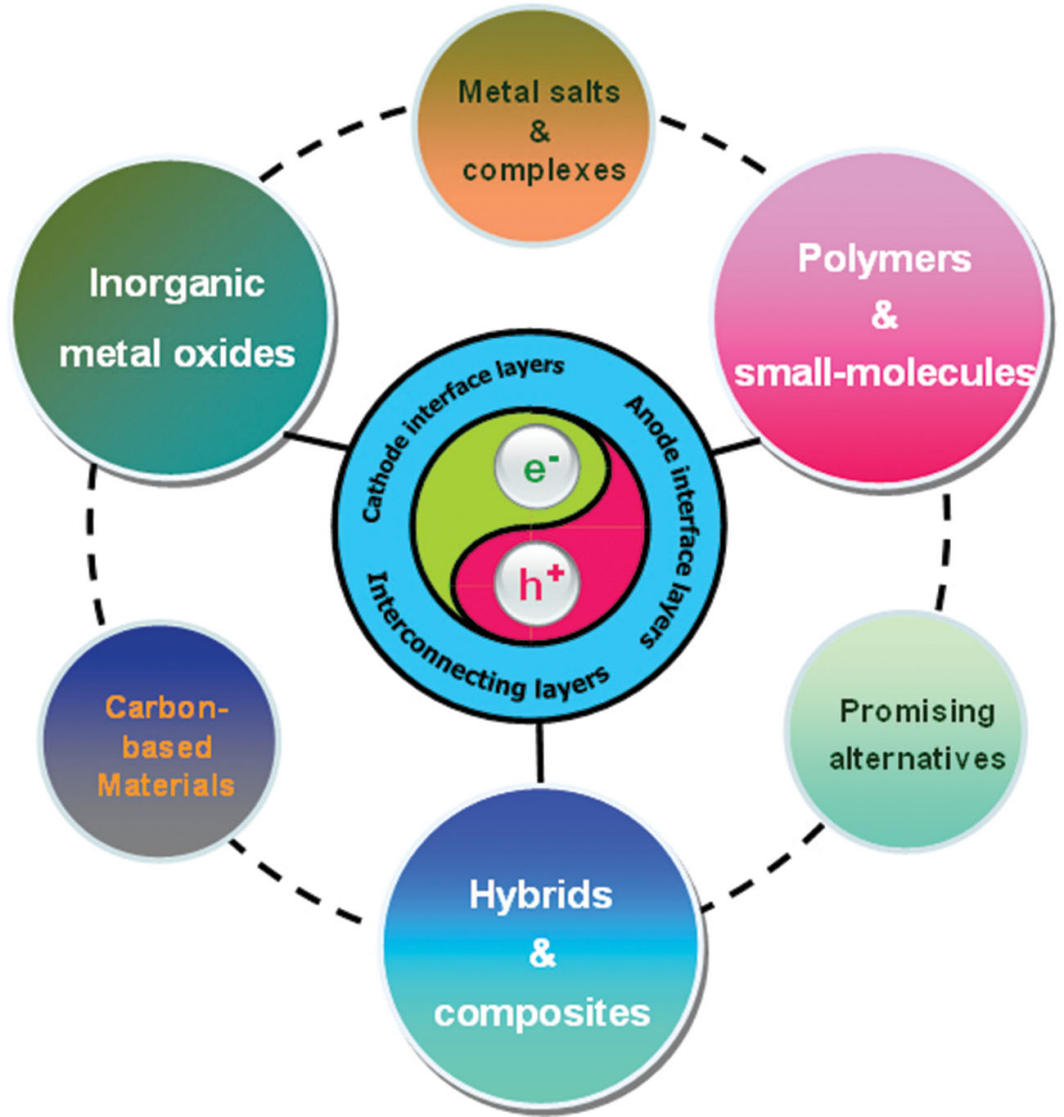
Schematic illustration of material categories for cathode interface layers, anode interface layers, and interconnecting layers used in OSCs.

It is known that the inevitable potential loss due to the energy level offset between the donor and acceptor materials in OSCs makes the electrode contacts crucial parameters to derive the net potential out of the BHJs.^34^ Therefore, it is required for OPV devices to form good Ohmic contacts at the interfaces between the active layer and electrodes for efficient charge extraction and transportation. The influence of electric contacts at different interfaces plays a fundamental role in affecting device performance such as open‐circuit voltage (*V*_OC_), short‐circuit current density (*J*_SC_), fill factor (*FF*), and the final PCE.^21^, ^35^, ^36^ Nowadays, a significant step for the achievement of high efficiency OSCs is engineering the driving force that realizes efficient extraction of holes and electrons to their respective electric contacts by adopting suitable interlayers as charge‐extracting constituents in OSCs, and also as charge recombination layers in tandem devices.^37^ Many efforts have been devoted to elucidate and engineer various interfacial layers in a wide range of single‐junction and tandem OSCs. The appropriate interfacial layers can be used to tune the energy level alignment at the electrode/active‐layer interfaces,^38^, ^39^, ^40^ to tailor the built‐in electric field,^38^, ^41^ to define the polarity of electrodes and improve charge selectivity,^21^, ^42^ to control surface energy by altering the morphology of the active layer,^43^ to introduce optical spacer and plasmonic effects to modulate light absorption in the active layer,^44^, ^45^, ^46^ and to improve interfacial stability between the active layer and electrodes.^47^, ^48^ All these interfacial layer functions are very beneficial to high efficiency and stable OSCs, which have been summarized in some excellent reviews.^49^, ^50^, ^51^ Note that the overall performance of OSCs highly depends on material structures and properties of various CILs, AILs and ICLs. However, studies on the interfacial material evolution and on understanding the material design principles as well as the relationships between structures and properties of interfacial layers have been very limited. Particularly, systematic discussions about ICLs used for tandem OSCs are seldom discussed. In this review, we shall exclude the contents which have already been covered by the available reviews, and instead we aim to discuss the latest developments on properties control of various interfacial layers and their influences on the device performance of both conventional and inverted OSCs. This is a significant undertaking because it comes at a time when a large number of novel electron/hole transporting materials are available for CILs, AILs and ICLs in high performance OSCs. As shown in **Figure** **2**, the presented materials of interfacial layers mainly include inorganic metal oxides, polymers/small‐molecules, carbon‐based materials, metal salts/complexes, organic‐inorganic hybrids/composites, and other alternatives. All these interfacial materials have a direct and essential impact on device efficiency and stability. Thus, we will present the structure adjustment and property optimization of these interlayers toward high efficiency OSCs. The related processing methods to control over the interfacial layers are also briefly discussed, along with their device performance. Our review intends to capture the latest and important aspects of interfacial layers in OPVs. But there may still have some missing publications of importance; if that is the case, we apologize to the authors of those papers.

Firstly, we systemically survey the design, fabrication, and application of various electron‐transporting materials as effective CILs for achieving high performance OSCs. Secondly, we will discuss the property control of various hole‐transporting materials as AILs for high performance OSCs. Thirdly, significant advances in tandem OSCs through developing several types of intermediate recombination materials as ICLs in both conventional and inverted tandem devices are highlighted. Interface engineering of all interfacial materials towards high performance single‐junction and tandem OSCs is comprehensively discussed, together with the structure–property relationships of interface layers as well as the device performance (especially for the efficiency and device stability). Finally, the review is ended with a brief concluding remark as well as some challenges and opportunities in the OPV field.

## 2 Electron‐Transporting Materials as CILs

For both conventional and inverted OSCs, CILs require low WFs to match with LUMO levels of acceptor materials for charge extraction, good electron‐transporting/hole‐blocking properties for charge‐transportation, and nice compatibility between cathodes and active layers for reducing interface defects and energy losses. Meanwhile, CILs should be highly transparent for light transmittance in inverted OSCs, and be stable to prevent from the diffusion of metal electrodes in conventional OSCs. To achieve excellent CILs for improving device performance, a great number of electron‐transporting materials, including semiconducting metal oxides, low WF metals and metal salts/complexes, polymers and small‐molecules, carbon‐based materials, hybrids/composites, and other emerging candidates have been developed. In this section, we mainly focus on new advances in property control of these CIL materials, together with a comprehensive discussion of their influences on device performance for both conventional and inverted OSCs. Device characteristics of some representative OSCs using various electron‐transporting CIL materials are summarized in **Table** **1**.

**Table 1:** Device characteristics of some representative OSCs with different CILs

OSC type	Cathode configuration	Active layer	Anode configuration	*V*_OC_ (V)	*J*_SC_ (mA cm^–2^)	*FF* (%)	PCE (%)	Ref.
Con.	Ba/Al	*p*‐DTS(FBTTh_2_)_2_:PC_71_BM	ITO/PEDOT:PSS	0.78	15.47	74.9	9.02	142
	ZnO/Al	PTB7:PC_71_BM	ITO/PEDOT:PSS	0.75	15.5	66	7.6	75
	TiO*_x_*/Al	PCDTBT:PC_71_BM	ITO/PEDOT:PSS	0.88	10.6	66	6.1	77
	Cs_0.5_MoO_3_/Al	PBDTDTTT‐S‐T:PC_71_BM	ITO/MoO_3_	0.68	16.08	66.9	7.32	58
	ZrAcac/Al	PBDTBDD:PCBM	ITO/PEDOT:PSS	0.89	14.25	72.7	9.23	154
	CsSt/Al	PTB7:PC_71_BM	ITO/PEDOT:PSS	0.73	16.05	61.3	7.16	148
	PFN/Ca/Al	PTB7:PC_71_BM	ITO/PEDOT:PSS	0.76	15.75	70.2	8.37	117
	PCCn6/Al	PTB7:PC_71_BM	ITO/PEDOT:PSS	0.73	15.19	73.2	8.13	120
	BCP/Ag	α‐6T/SubNc/SubPc	ITO/PEDOT:PSS	0.96	14.55	61.0	8.40	135
	PDINO/Al	PTB7‐Th:PC_71_BM	ITO/PEDOT:PSS	0.80	15.45	67.6	8.36	136
	Phen–NaDPO/Al	PTB7:PC_71_BM	ITO/PEDOT:PSS	0.75	16.81	68	8.56	137
	MSAPBS/Al	PTB7:PC_71_BM	ITO/PEDOT:PSS	0.76	19.25	68	10.02	139
	C_60_–N/Ag	PTB7‐Th:PC_71_BM	ITO/PEDOT:PSS	0.78	16.83	71.4	9.35	160
	GO/TiO*_x_*/Al	PCDTBT:PC_71_BM	ITO/PEDOT:PSS	0.88	12.40	68	7.50	168
	POM 6/Al	PCDTBT:PC_71_BM	ITO/MoO*_x_*	0.88	12.7	66	7.4	190
Inv.	ITO/ZnO	PIFTBT8:PC_71_BM	MoO_3_/Ag	1.04	9.74	50.1	5.05	48
	ITO/patterned ZnO	PTB7‐Th:PC_71_BM	MoO*_x_*/Al	0.78	19.47	66.9	10.1	10
	ITO/TiO_2_	PBDTTT‐C‐T:PC_71_BM + Au NPs	MoO_3_/Ag	0.76	18.39	62.9	8.79	84
	ITO/N‐TiO*_x_*	PTB7‐Th:PC_71_BM	MoO_3_/Ag	0.79	15.50	72	8.82	87
	ITO/SnO_2_	PBDTT‐DPP:PCBM	MoO_3_/Al	0.73	11.74	61.2	5.24	93
	ITO/Zn_1–*x*_Mg*_x_*O	PTB7:PC_71_BM	MoO_3_/Ag	0.74	16.78	67.0	8.31	57
	ITO/IZO	PTB7‐Th:PC_71_BM	MoO_3_/Ag	0.79	16.42	70.2	9.11	26
	ITO/AZO	PTB7‐Th:PC_71_BM	MoO*_x_*/Ag	0.80	17.7	70.7	9.94	109
	ITO/Al:MoO_3_	PCDTBT:PC_71_BM	MoO_3_/Al	0.88	10.88	70.7	6.77	98
	ITO/V_2_O*_x_*:Cs	PBDTDTTT‐S‐T:PC_71_BM	V_2_O*_x_*/Ag	0.63	15.81	61.0	6.08	112
	ITO/PFN	PTB7‐Th:PC_71_BM	MoO_3_/Al	0.83	17.43	73.8	10.61	12
	ITO/PFEN‐Hg	PTB7:PC_71_BM	MoO_3_/Al	0.74	17.37	71.2	9.11	125
	ITO/FTBTF‐N	PTB7:PC_71_BM	MoO_3_/Al	0.74	17.23	72.1	9.22	138
	ITO/C_60_–SB	PTB7‐Th:PC_71_BM	MoO_3_/Ag	0.75	18.24	66.0	9.08	164
	ITO/PEG–TiO*_x_*	PTB7‐Th:PC_71_BM	MoO_3_/Ag	0.79	17.40	65.6	9.05	27
	ZnO:P(VDF–TrFE)	PTB7:PC_71_BM	MoO_3_/Ag	0.75	14.21	76.5	8.15	176
	ITO/ZnO:PBI‐H	PTB7‐Th:PC_71_BM	MoO_3_/Al	0.82	17.69	72.9	10.59	177
	ITO/ZnO/PEI	PTB7:PC_71_BM	MoO_3_/Ag	0.73	17.27	70.1	8.88	182
	ITO/ZnO/[BMIM]BF_4_	PTB7‐Th:PC_71_BM	MoO_3_/Ag	0.78	17.70	73.5	10.15	181
	ITO/ZnO–C_60_	PTB7‐Th:PC_71_BM	MoO_3_/Ag	0.80	15.73	74.3	9.35	25
	ITO/FPI‐PEIE	PBDTT‐TT:PC_71_BM	MoO_3_/Ag	0.80	16.15	72	9.62	185

Note: Con. represents the conventional OSC; Inv. represents the inverted OSC.

### 2.1 Metal Oxides

Among the family of electron‐transporting materials, *n*‐type metal oxides with deep‐lying energy levels are currently predominant CIL materials in single‐junction and tandem OSCs. OSCs based on metal oxide CILs exhibit high performance which can be contributed to the fact that the metal oxide CILs have salient features of ambient stability, good solution processability, high optical transparency, and excellent capability to extract/transport electron carriers. To date, effective CILs for OSCs include binary oxides (such as ZnO, TiO*_x_*, Nb_2_O_5_, and SnO*_x_*),^52^, ^53^, ^54^, ^55^ and newly emerged ternary oxides (such as Al‐doped ZnO, Mg‐doped ZnO, and Cs‐doped metal oxides).^56^, ^57^, ^58^

#### 2.1.1 Zinc Oxide (ZnO)

As an inorganic *n*‐type semiconductor, ZnO is one of the best choices in metal oxide CIL materials due to its features such as low cost, easy synthesis, non‐toxicity, high stability, and unique optical/electronic properties.^59^ Generally, ZnO materials have a low WF of ≈4.30 eV, which offers a descent energy level to reduce WF of ITO or metal electrodes, and to match with LUMO levels of various fullerene‐based acceptors such as [6,6]‐phenyl‐C61‐butyric acid methyl ester (PCBM), PC_71_BM, and indene‐C_60_ bis‐adduct (ICBA), etc. The optical transparency, mobility, and interfacial properties of ZnO CIL materials could be tuned with their variations in crystalline structures, film morphologies, surface energy and compositions, film thickness, and defects, etc. which greatly rely on the processing conditions.^60^, ^61^, ^62^, ^63^, ^64^, ^65^ Correspondingly, the device performance of OSCs using various ZnO materials as CILs are different. Therefore, property control of ZnO CIL materials to balance their transmittance, electron mobility and interfacial properties, is critical for high performance OSCs.

So far, various methodologies have been used to fabricate and control over ZnO CIL materials for improving efficiency and stability of OSCs. The synthetic strategies include electrochemical deposition,^66^ sol–gel processing,^52^ hydrothermal growth,^67^ nanoparticle approaches,^68^ atomic layer deposition (ALD),^69^ and so on. Among them, the sol–gel strategy is a very promising way to achieve high performance ZnO CILs using some zinc salts as precursors. Despite this method requires high temperature post‐annealing to complete the hydrolysis reaction, it is still a good way to fabricate ZnO films as the bottom CILs without any additional steps. Earlier researches with sol–gel ZnO CILs mostly focused on the poly(3‐hexylthiophene) (P3HT)‐based system which exhibited PCEs less than 5%.^59^ Replacing P3HT in the BHJ with a narrower bandgap polymer poly[[9‐(1‐octylnonyl)‐9H‐carbazole‐2,7‐diyl]‐2,5‐thiophenediyl‐2,1,3‐benzothiadiazole‐4,7‐diyl‐2,5‐thiophenediyl] (PCDTBT), increased PCEs over 6% were then achieved for the inverted OSCs using low‐temperature sol–gel ZnO as the CILs.^70^ Applying the sol–gel ZnO film to a new polymer system (PffBT4T‐2OD), PCEs up to 10.8% were demonstrated, representing a record high efficiency for single‐junction OSCs to date.^28^

**Figure 3:**
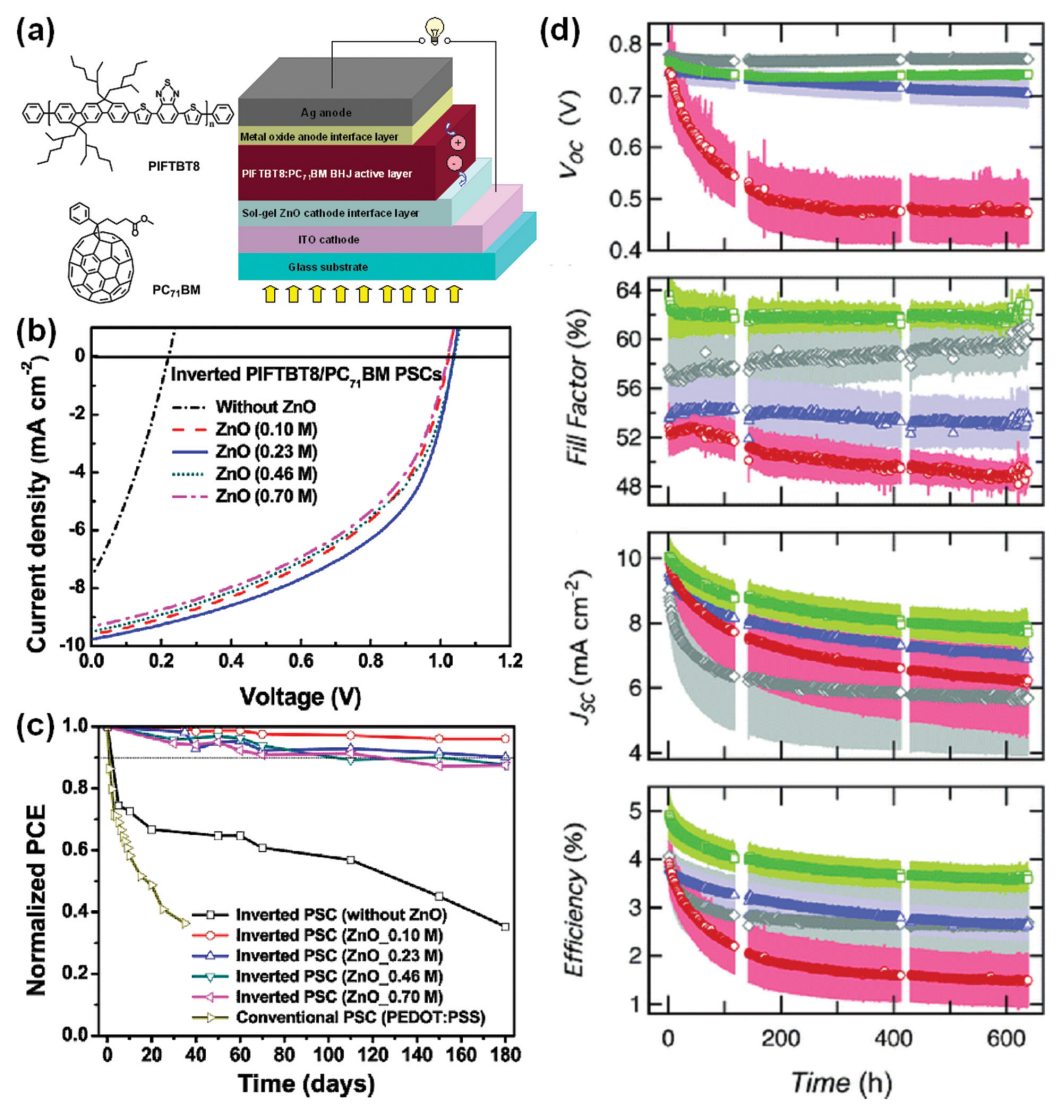
a) Molecular structures of PIFTBT8 and PC_71_BM, and a schematic device with the ZnO CIL. b) Current density‐voltage (*J−V*) characteristics under AM 1.5G irradiation (100 mW cm^−2^), and c) Device stability of the inverted OSCs with ZnO CILs derived from controlled precursor solutions. Reproduced with permission.^48^ Copyright 2013, American Chemical Society. d) Average performance parameters of devices with ZnO CILs derived from ZnAc (red circles), deZn (blue triangles), PA‐modified ZnAc (green squares), and PA‐modified deZn (gray diamonds): *V*_OC_, *FF*, *J*_SC_, and PCE, all as a function of time exposed to the degradation solar simulator. Reproduced with permission.^71^ Copyright 2015, Royal Society of Chemistry.

Note that the device performance of OSCs with sol–gel ZnO strongly depends on the use of precursors and annealing treatments. By controlling concentrations of ZnO precursor sols, several amorphous ZnO films with high transparency and electron mobility were made by Yin et al.^48^ These controlled ZnO films were demonstrated as effective CILs for efficient and long‐term stable OSCs with high *V*_OC_s of 1.00–1.06 V. The inverted OSCs were based on a BHJ of indenofluorene‐containing copolymer (PIFTBT8) and PC_71_BM (**Figure** **3**a). Incorporation of the optimized ZnO CIL can minimize interfacial energy losses and improve electron extraction/collection, leading to great increases in *V*_OC_ from 0.22 to 1.04 V and *J*_SC_ from 7.61 to 9.74 mA cm^−2^, and subsequently a remarkable improvement in PCE from 0.51% to 5.05% (Figure 3b). More significantly, all the devices based on the sol–gel ZnO CILs showed much better stability than that of devices without the ZnO CIL and the conventional OSCs (Figure 3c). The best device with the sol–gel ZnO maintained a PCE of 4.55% (>90% of its initial efficiency) and a *V*_OC_ of 1.05 V over half a year, demonstrating excellent long‐term stability for the inverted OSCs. Recently, Olson et al. demonstrated that inverted OSCs using diethylzinc (deZn)‐derived ZnO CILs are more stable under long‐term illumination than the devices using zinc acetate (ZnAc)‐derived ZnO CILs (Figure 3d).^71^ Furthermore, when the ZnO CIL was made from a dipolar phosphonic acid (PA)‐modified ZnAc, the corresponding device exhibited improved device performance and stability compared with the device based on the unmodified ZnAc‐derived ZnO. However, this strategy is not suitable for the deZn‐based ZnO CILs. These results demonstrate that the interface engineering of sol–gel ZnO CILs is an important strategy to develop high efficiency and long‐term table OSCs.

**Figure 4:**
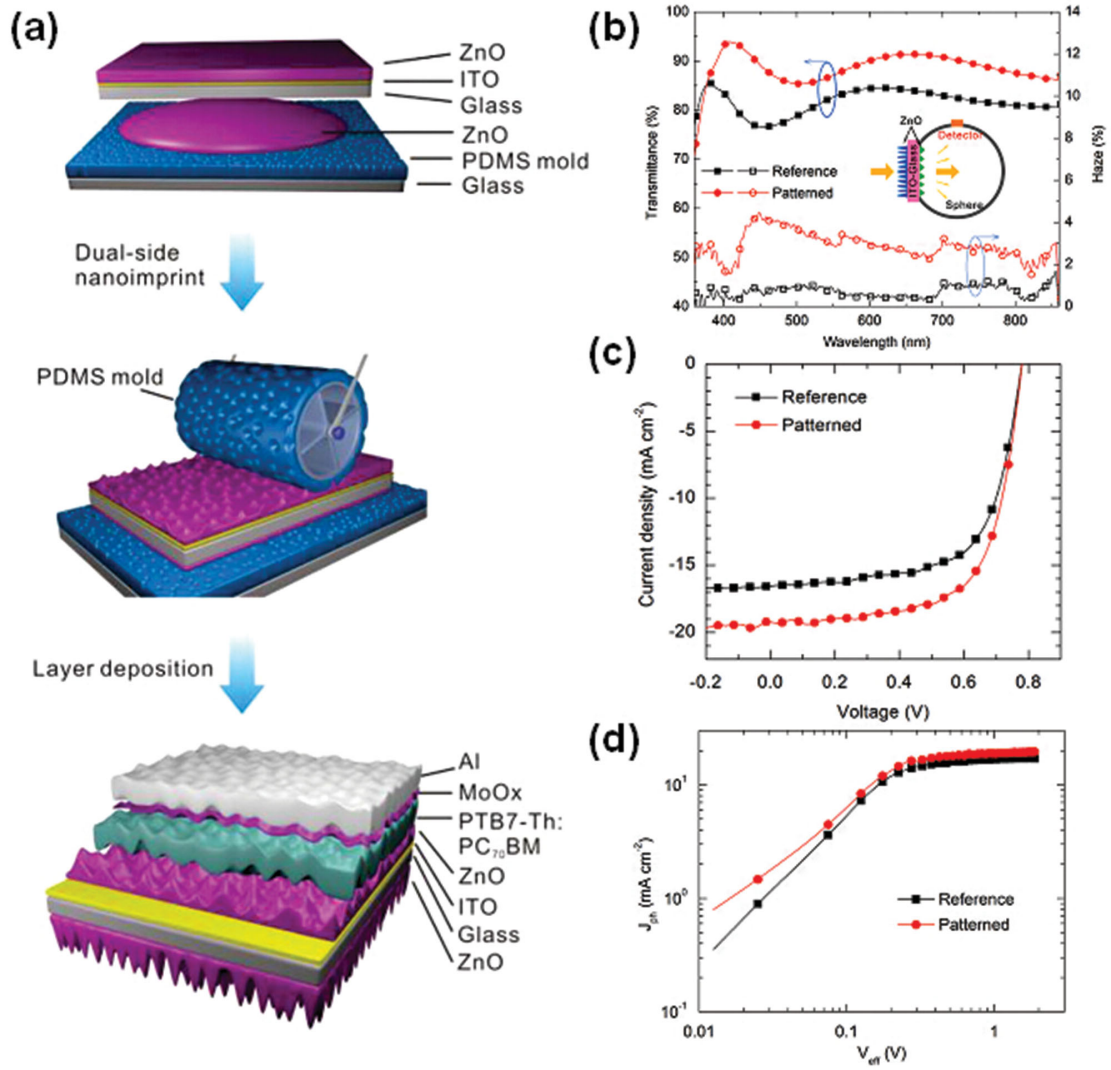
a) Schematic illustration of a preparation process for OSCs containing dual‐sided nanoimprinted DNAs. b) Total transmittance and haze values of ZnO/ITO glasses without and with DNA‐patterns. The inset depicts the optical measurement configuration using an integrating sphere. c) *J*–*V* and d) *J*_ph_–*V*_eff_ characteristics for the reference and patterned OSCs under AM 1.5G illumination. Reproduced with permission.^10^

Recently, a patterned sol–gel ZnO CIL was developed by Tang and co‐workers for inverted PTB7‐Th:PC_71_BM‐based devices.^10^ **Figure** **4**a depicts a schematic fabrication process for the OSC incorporating dual‐side DNA patterning of ZnO layers by a rolling nanoimprinting technique using polydimethylsiloxane (PDMS) molds. As shown in Figure 4b, the patterned ZnO/ITO glass, exhibits an increase in the transmittance over 350–800 nm in comparison with the regular ZnO/ITO glass, which is attributed to the enhancement of light anti‐reflection and transmission. It is evident that light scattering significantly contributes to the total transmission, where the average haze (Figure 4b) for ZnO/ITO–glass improved from 0.35% to ≈2.65% over the visible spectrum. The great transmittance enhancement resulted in the self‐enhanced light absorption in OSCs with the patterned ZnO on ITO–glass. Meanwhile, the superior photocurrent density‐effective voltage (*J*_ph_–*V*_eff_) characteristics (Figure 4d) from the patterned device identify the higher charge extraction property in the patterned OSCs due to the optimized contact between the pattered ZnO and the active layer. These benefits of self‐enhanced absorption resulted in the performance enhancement of the OSCs with patterned ZnO CILs. Accordingly, compared to the device with a flat ZnO CIL, an 18% increase in photocurrent and an improved PCE from 8.46% to 10.1% was achieved without sacrificing the charge transporting properties (Figure 4c). These patterned ZnO CILs may have good potential for other high efficiency OSCs.

Besides the sol–gel ZnO, the use of ZnO nanoparticles (NPs) is another promising pathway for enhancing device performance of OSCs. ZnO NPs can be directly spin‐coated from solution onto active layers or an ITO electrode, which makes them a good candidate for efficient CILs in both conventional and inverted devices. With the development of active layer materials and processing technologies, the PCEs of OSCs using ZnO NPs as CILs rapidly increased to 7–8% in recent years.^72^, ^73^, ^74^, ^75^ For example, by the insertion of solution‐processed ZnO NPs as an optical spacer, the performance of conventional OSCs was greatly improved even without an increase in light absorption.^75^ The device performance enhancement was due to the electron‐extracting/hole‐blocking properties of ZnO NPs in combination with a reduction of contact resistance and charge recombination at the Al/BHJ interface. The morphology of ZnO CILs is a crucial factor to obtain high efficiency devices. By modification with ethanolamine, the cluster free ZnO NP solution could be prepared as CILs with improved film morphology and improved contacts with respect to untreated ZnO NPs. Through optimizing optical, morphological and electronic properties of ZnO NPs‐based CILs, the final PCE was improved from 5.8% to 7.6% in the conventional PTB7:PC_71_BM‐based devices.

Since sol–gel derived ZnO always requires a high‐temperature annealing, the ZnO NPs are a good choice of materials as CILs for inverted OSCs due to its low temperature processing capability. For example, using the ZnO NPs as the CIL, inverted devices exhibited a high PCE of 7.4% for the BHJ of poly(dithieno[3,2‐b:2′,3′‐d]germole thieno[3,4‐c]pyrrole‐4,6‐dione) (PDTG‐TPD):PC_71_BM.^73^ The surface defects or trap sites in the ZnO NP layers can be passivated by a UV–ozone (UVO) treatment. The reduction of defects in the UVO‐treated ZnO NPs was confirmed by a loss of emission from the defect, and an increased carrier lifetime. As a result, inverted OSCs based on the CIL of UVO‐treated ZnO NPs, delivered a significant enhancement of *J*_sc_ and a final improved PCE of 8.1%.

The greatly enhanced device performance for OSCs with ZnO CILs is mainly due to their favorable electronic and optical properties, which make ZnO an ideal interfacial layer material for OPV devices. At the same time, ZnO materials can be easily processed via a solution way followed by relatively low‐temperature annealing, making ZnO fully compatible with roll‐to‐roll processing onto flexible substrates in the future. However, it should be noted that the fixed characteristics of binary ZnO and the existed defects within the ZnO materials still need to be resolved by some strategies such as chemical/physical doping, and interfacial engineering, etc.

#### 2.1.2 Titanium Oxide

Titanium oxide (TiO_2_ or TiO*_x_*) is another *n*‐type metal oxide CIL material because it has good optical transparency, relatively high electron mobility, and environmental stability. By solution processing from a sol–gel or spin‐coating of TiO*_x_* NPs, TiO*_x_* films were fabricated as effective CILs for both conventional and inverted OSCs.^53^, ^76^, ^77^, ^78^, ^79^ When incorporated into conventional OSCs, titanium oxide can serve as an optical spacer to redistribute the light intensity within the active layer to enhance light absorption, and can act as an electron‐transporting/hole‐blocking layer to improve charge collection.^77^, ^78^ As a result, the conventional PCDTBT:PC_71_BM‐based device using the sol–gel TiO*_x_* CIL showed an increased PCE of 6.1% with an internal quantum efficiency approaching 100%.^77^ The sol–gel TiO*_x_* CIL was introduced between the active layer and Al cathode to act as a shielding and scavenging layer, which prevented the oxygen/humidity from penetrating the organic active layer.^76^ Thus the ambient lifetime of the conventional devices with the TiO*_x_* CIL was enhanced by two orders of magnitude compared to those without the TiO*_x_* CIL. Similarly, incorporating TiO_2_ NPs as a CIL in conventional OSCs, both the PCE and stability were improved.^78^ The P3HT:PCBM‐based devices with a TiO_2_ NPs CIL achieved an increased PCE of 4.24%, while maintained 80% of the original PCE after 200 h storage in air, much better than the devices without such a CIL.

When titanium oxide is inserted between ITO and the active layer, it can work as a universal CIL in inverted OSCs by aligning energy levels and extracting/transporting electrons. As to the common sol–gel‐derived TiO*_x_*, it is found that titania precursors and annealing temperatures can significantly affect the film structures and optoelectronic/interfacial properties of the resulting TiO*_x_* CILs, ultimately influencing the device performance.^79^, ^80^ For instance, substituting isopropyl ligands of titanium isopropoxide with 2‐methoxyethanol led to TiO*_x_* CILs that required a shorter illumination time to fill shallow electron traps.^80^ This decrease in trap density of CILs led to OSCs with shorter saturation time and better performance. Using the sol–gel TiO*_x_* CIL, increased PCEs of 4.65% and 5.5% were achieved for inverted OSCs based on the P3HT and PCDTBT system, respectively.^79^, ^81^ The sol–gel TiO*_x_* CILs can also be used to establish stable inverted devices with only 3.67% reduction in PCE over 2160 h of storage.^82^ Recently, a low‐temperature solution derived TiO*_x_* CIL was developed to greatly enhance the efficiency and stability of inverted OSCs, where the normalized PCE could retain over 90% after 120 days storage in air.^27^ Control over the film thickness and morphology of TiO*_x_* CILs by a facile electro‐deposition was also used to enhance and optimize device performance of inverted OSCs.^83^ In addition, TiO_2_ NPs were similarly designed as effective CILs to reduce WF of ITO and facilitate electron‐collection, where high PCEs up to 8.79% were achieved for the inverted OSCs with a polymer donor poly{[4,8‐bis‐(2‐ethylhexyl‐thiophene‐5‐yl)‐benzo[1,2‐b:4,5‐b′]­dithiophene‐2,6‐diyl]‐*alt*‐[2‐(2′‐ethyl‐hexanoyl)‐thieno[3,4‐b]thiophen‐4,6‐diyl]} (PBDTTT‐C‐T) in the BHJ.^84^

It should be noted that many defects/trap sites are existed in TiO*_x_* layers, giving rise to a Schottky barrier at the high WF‐metal/metal‐oxide interface or energy level mismatching at the metal oxide/BHJ layer interface. These problems hinder the effectiveness of TiO*_x_* as CILs and thus reduce the device performance. A strategy of light soaking with UV radiation is always needed to perform on the TiO*_x_* layers to reduce their oxygen defects, decrease the resistance, and increase the carrier density, finally removing the S‐shaped *J–V* characteristics and recovering the device performance.^85^, ^86^, ^87^ As an alternative to light‐soaking, the chemical doping of TiO*_x_* by nitrogen was recently used to increase the carrier density in TiO*_x_* CILs and significantly reduce the WF of ITO from 4.80 to 4.20 eV, leading to a great increase in PCE from 2.13% to 8.82%.^87^ Additionally, the use of thin metal oxides or polymer layers to modify TiO*_x_* can also effectively passivate the surface trap states of TiO*_x_*, and subsequently improve the device performance of OSCs.^27^, ^88^, ^89^

#### 2.1.3 Niobium Oxide

Similar to ZnO and TiO*_x_*, wide‐bandgap niobium oxide (Nb_2_O_5_ or NbO*_x_*) materials have high transparency at visible wavelengths and inherently *n*‐type characteristics. Thereby they can act as electron‐transporting CILs for OSCs.^54^, ^90^, ^91^ As a new candidate for binary oxide CILs in OPVs, Nb_2_O_5_ CILs were prepared by sol–gel methods for improving device performance of both conventional and inverted OSCs,^54^, ^92^ A drawback of Nb_2_O_5_ lies in the fact that its conduction band (≈3.7 eV) is higher than the LUMO level of PCBM, which could reduce its effectiveness of electron‐extraction in fullerene‐based OSCs. Whereas solution‐processed amorphous NbO*_x_* CILs showed a larger conduction band value (4.0 eV), which provided a better energy matching to improve the performance in P3HT:PCBM devices.^91^ Accordingly, the adequate energy level turning of niobium oxide materials is needed in the future.

#### 2.1.4 Tin Oxide

Tin oxide (SnO_2_ or SnO*_x_*) is another promising wide‐bandgap metal oxide. The higher intrinsic mobility of tin oxide compared to other *n*‐type oxides, offers its advantages in the efficient carrier transport. Recently, solution‐processed SnO*_x_* films and SnO_2_ NPs were developed as CILs for inverted OSCs.^55^, ^93^, ^94^ Using tetrakis(diethylamino)tin as a precursor, the room‐temperature processed SnO*_x_* exhibited a low WF of 4.1 eV comparable to that of TiO*_x_* (≈4.0 eV).^55^ Inverted OSCs using this SnO*_x_* CIL afforded a PCE similar to that of TiO*_x_* CIL‐based OSCs. Interestingly, unlike the devices with TiO*_x_*, the OSCs based on the SnO*_x_* CILs without encapsulation were very stable even upon heat treatment in humid air. Designing SnO*_x_* CILs via ALD at the temperature of 80 °C, the necessity of UV activation that comes with the use of TiO*_x_* and ZnO–based CILs can be avoided.^94^ When used for inverted OSCs, the SnO*_x_* can act as a universal “light‐soaking” free material for electron extraction, delivering a much higher PCE of 5.7% compared to 0.4% for OSCs using TiO*_x_* without UV activation. This result can be attributed the fact that the SnO*_x_* allowed for efficient barrier‐free electron extraction even without UV activation, and its WF of 4.2 eV remained constantly before and after UV illumination. By contrast, ZnO and TiO*_x_* substantially lowered their WFs upon UV illumination. Precise control over surface structures of tin oxide CILs is also critical to the OSC performance. A solvothermal way to stabilize gelled dispersions of SnO_2_ NPs with diameters of 2−4 nm was designed, allowing the generation of smooth SnO_2_ CILs.^93^ The small particle size, smooth film morphology, and excellent electron‐transporting properties of the nanocrystalline SnO_2_ offered an effective foundation upon which to grow high quality polymer/fullerene active layers. As a result, a dramatically improved PCE of 5.24% was achieved for the inverted OSCs based on poly{2,6′‐4,8‐di(5‐ethylhexylthienyl)benzo[1,2‐b;3,4‐b]dithiophene‐*alt*‐5‐dibutyloctyl‐3,6‐bis(5‐bromothiophen‐2‐yl)pyrrolo[3,4‐c]pyrrole‐1,4‐dione} (PBDTT‐DPP):PCBM. By contrast, the devices using extended SnO_2_ and bulk SnO_2_ as CILs showed much lower *V*_OC_, *FF* and PCE values. The presence of large particles and defects in the extended/bulk SnO_2_ suspension prevents the formation of good CILs that are necessary to minimize shunting pathways and leakage currents for achieving high‐efficiency devices. All these results demonstrate that tin oxide materials are a new class of CILs for high performance OSCs. Considering the salient properties of tin oxides, it is expected that the design of novel tin oxide‐based interlayers may pave a promising way towards OSCs with high PCEs and long‐term stability.

#### 2.1.5 Alumina (Al_2_O_3_) and Zirconia (ZrO_2_)

Besides the semiconducting binary oxides, low WF Al_2_O_3_ or ZrO_2_ insulating nanolayers can also be used to reduce the WF of cathode and promote charge collection in OSCs. With the ALD method or the UV–O_3_ treatment on Al thin‐films, Al_2_O_3_ layers were designed as effective CILs to improve the OSC efficiency and stability.^95^, ^96^ Nevertheless, the performance of these inverted devices was sensitive to the Al_2_O_3_ CILs, which need a control of ultrathin thickness and UV activation on them to enhance their electrical conductivity. Recently, a low temperature and solution‐processing route was used to tune the properties of Al_2_O_3_ CILs.^97^ The WFs of Al_2_O_3_ CILs were tuned by controlling the annealing temperature. With an optimized Al_2_O_3_ CIL (150 °C) having a low WF of 3.89 eV, the inverted OSCs exhibited enhanced diode characteristics and achieved a 20% higher PCE than that with the ZnO CIL. The improved performance is due to the fact that a higher transmittance of the Al_2_O_3_ CIL allowed the inverted OSCs absorbing more incident photons than the ZnO‐based devices. On the other hand, the Al_2_O_3_ CIL has a larger bandgap, which makes it more effective in blocking holes and reducing charge recombination. Recently, thermally ALD‐processed Al_2_O_3_ or ZrO_2_ nanolayers on TiO_2_ films were developed as versatile CILs for electron‐extraction in a wide range of polymer solar cells, resulting in PCEs up to 7.1%.^89^

#### 2.1.6 Ternary Metal Oxides

As discussed above, the binary oxides have been widely investigated as electron‐transporting materials for achieving high performance OSCs. However, their major limitations as CILs are their fixed characteristics such as bandgaps, energy levels, transmittance, and conductivity, etc. These fixed characters indicate the inflexibility in general applications for OSCs due to the variable energy levels of donor/acceptor materials. To overcome these weaknesses, designs of ternary metal oxides with tunable compositions and properties, can be significant strategies to improve OSC performance. Using *n*‐type doping in binary oxides, a series of ternary metal oxide electron‐transporting materials were developed as CILs.^56^, ^57^, ^58^, ^98^, ^99^, ^100^ When they served as CILs in inverted OSCs, improved PCEs and device stability were demonstrated, providing a very promising strategy to develop high performance OSCs toward practical utilization.

**Figure 5:**
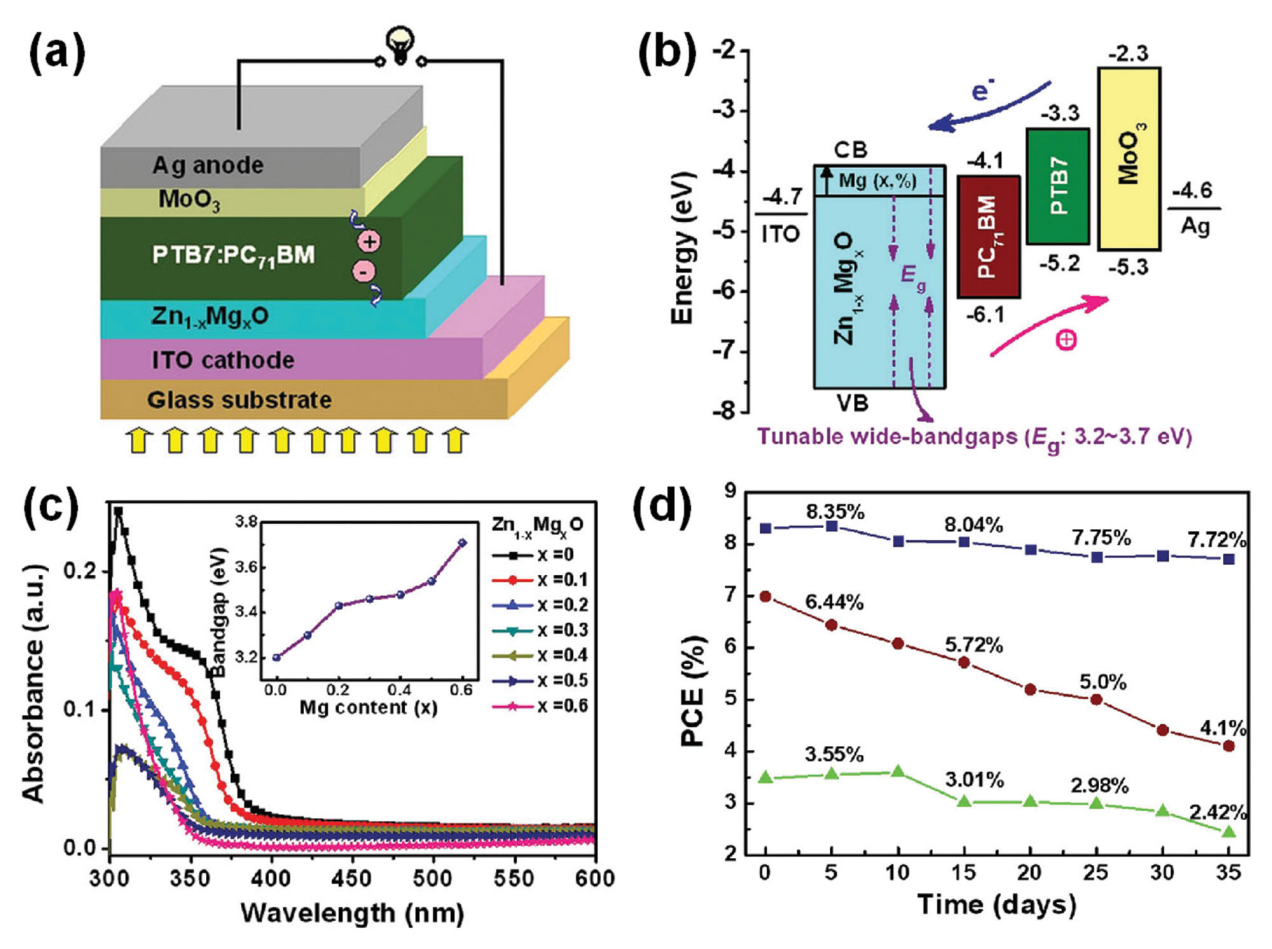
a) A schematic device structure based on the ZMO CIL. b) Energy levels of the components in the OSCs with various ZMO as the CIL. c) Optical absorption spectra of ZMO films. The inset shows an increase in the bandgap of ZMO films. d) Device stability of three types of devices: the best ZMO CIL‐based inverted OSC (▪), the PEDOT:PSS‐based conventional OSC (●), and the inverted OSC without a CIL (▴).Reproduced with permission.^57^

It is known that the intrinsic ZnO and TiO*_x_* have major absorption in the UV region and thus limits the devices absorbing more incident photons in that region. Therefore, designing larger bandgap ternary oxides is desired for high performance OSCs. Recently, bandgap tunable Zn_1–*x*_Mg*_x_*O (ZMO) metal oxides were proposed by Yin et al. as a novel class of CILs for enhancing efficiency and stability of OSCs (**Figure** **5**).^57^ By Mg doping in ZnO, solution‐processed ZMO CILs showed tunable bandgaps, WFs and energy levels depending on the amount of Mg doping, thereby enabling to tune their transmittance, charge‐collection, and interfacial properties for a better device performance (Figure 5b–d). The inverted PTB7:PC_71_BM OSCs based on ZMO CILs exhibited high PCEs up to 7.83% (8.31–8.35% with further optimization), which is much better than those of the control devices without a CIL (PCE = 3.48%) or with a ZnO CIL (PCE = 7.11%). Meanwhile, all the inverted OSCs using ternary ZMO CILs displayed good stability, which maintained over 93% of initial values after storage in air for 5 weeks. The best device based on the ZMO retained high PCEs of 7.72% after 35 days and 7.46% over 4 months, much better than those of the inverted OSCs without a CIL and the conventional OSCs (Figure 5d). The results open an efficient pathway for the design of highly efficient and stable OSCs using multielement semiconductors with tunable bandgaps as CILs.

It is known that intrinsic ZnO and TiO*_x_* have relatively low conductivities and thus are limited in the film thickness when used as CILs. To overcome this restriction, a viable route is to dope them with high conductivity main group‐III/IV elements such as Al, Ga, In or Sn. Thus various ternary metal oxides as CILs were designed, including Al‐doped ZnO (AZO),^56^, ^101^, ^102^ Ga‐doped ZnO (GZO),^103^, ^104^ In‐doped ZnO (IZO),^105^ and Sn‐doped ZnO (ZTO).^106^ The conductivity of AZO is three orders of magnitude higher than that of ZnO.^101^ Thereby the thickness of AZO CILs can be increased up to 100 nm and even 680 nm, while the PCEs of the inverted OSCs are still comparable to the devices with thin AZO CILs.^101^, ^102^ By contrast, the OSCs using 100 nm ZnO CILs suffered from remarkably increased series resistance (*R*_S_) values and reduced PCEs. Similarly, with GZO CILs up to 200 nm thickness or IZO CILs up to 160 nm thickness, very small impacts on their device performance were found due to the greatly enhanced conductivities of GZO and IZO compared to ZnO.^103^, ^105^ Note that the device performance strongly depends on the dopant concentrations, precursors, annealing conditions, surface morphologies and roughnesses of ternary oxide CILs.^56^, ^102^, ^104^, ^107^, ^108^ Thus all these factors should be systemically evaluated for balancing the interfacial/optoelectronic properties of ternary oxide CILs when fabricating OPV devices. Incorporating the optimized ternary oxides as CILs, better performance of OSCs were observed for all the AZO, IZO, GZO, and ZTO CILs because of their reduced recombination at the interfaces, improved electron‐transport/hole‐blocking properties with respect to the standard ZnO CILs.^26^, ^104^, ^106^, ^109^ For example, the In doping in ZnO improved the surface conductivity by a factor of 567 (from 0.015 to 8.51 S cm^−1^) and enhanced electron mobility in the vertical direction by a factor of 115 (from 8.25 × 10^−5^ to 9.51 × 10^−3^ cm^2^ V^−1^ s^−1^).^26^ The resultant inverted OSCs (PTB7‐Th:PC_71_BM) using the IZO CIL exhibited an improved PCE of 9.11% in relative to the devices with ZnO (PCE = 8.25%). Very recently, low‐temperature (125 °C) solution‐processed AZO NPs were also demonstrated as electron‐transporting/hole‐blocking CILs for a broad range of polymer:fullerene inverted OSCs, yielding high PCEs over 10% (8%) on glass (plastic) substrates.^109^ Additionally, the use of Al to dope MoO_3_ can form ternary Al:MoO_3_ CILs with high transmittance and tunable WFs, which can be finely adjusted by controlling Al contents in the CILs.^98^ Correspondingly, the interfacial property at CIL/BHJ interfaces can be optimized to reduce the recombination loss and to facilitate electron extraction/collection, resulting in inverted single‐junction and tandem PCDTBT:PC_71_BM devices with PCEs of 6.77% and 7.31%, respectively.^98^, ^110^

Alkali metal elements such as Li and Cs were incorporated into binary oxides to design ternary metal oxide CILs such as Li‐doped ZnO (LZO), Cs‐doped ZnO, Cs‐doped TiO_2_, Cs‐doped molybdenum oxide (MoO_3_ or MoO*_x_*), and Cs‐doped vanadium oxide (V_2_O_5_ or V_2_O*_x_*).^58^, ^72^, ^111^, ^112^ The suitable Li or Cs doping can enhance the conductivity and improve electron‐transporting of ZnO or TiO_2_ CILs in both single‐junction and tandem OSCs, leading to a record high PCE up to 11.83% with good ambient stability.^31^, ^72^, ^111^ On the other hand, the Cs doping approach can convert hole‐transporting binary oxides into electron‐transporting ternary oxides, where a wide range of controllable WF adjustments, energy levels, and electrical properties can be realized by tuning the ratio of Cs in ternary oxides. Using a solution processing way, over 1.1 eV WF tuning of MoO_3_ and V_2_O_5_ was realized by Cs intercalation, making the Cs_0.5_MoO_3_ and CsV_2_O_5_ function as effective CILs for developing conventional OSCs with high PCEs of 7.32% and 7.49%, respectively, based on poly[(((2‐hexyldecyl)sulfonyl)‐4,6‐di(thiophen‐2‐yl)thieno[3,4‐b]thiophene‐2,6‐diyl)‐*alt*‐(4,8‐bis((2‐ethylhexyl)oxy)benzo[1,2‐b:4,5‐b′]dithiophene‐2,6‐diyl)] (PBDTDTTT‐S‐T):PC_71_BM.^58^ Similarly, with the doping of Cs in MoO*_x_* and V_2_O*_x_*, continuous variation of WFs can be over 1 eV for the Cs‐intercalated metal oxides.^112^ When used for OSCs, both conventional and inverted devices showed good performance, where PCEs of 6.08% and 7.44% were achieved, respectively for the best inverted and conventional OSCs (PBDTDTTT‐S‐T:PC_71_BM) using the V_2_O*_x_*:Cs CIL. The success of ternary Cs‐intercalated metal oxides offers a facile pathway to obtain a large continuous WF tuning and structure adaptability for optoelectronic devices. Additionally, using the sol–gel processing or co‐sputtering, the Zn or Cd element can be doped into TiO_2_ or TiO*_x_* films to optimize energy alignments, promote fast electron‐transport, and improve light transmittance of the ternary oxides, finally leading to efficient CILs for high performance OSCs.^113^, ^114^, ^115^

It is found that the ternary metal oxides can be used as an alternative to other widely known CIL materials to improve the performance and stability of OSCs. The latest developments could be applied to other electronic devices as well.

### 2.2 Polymers and Small‐Molecules

**Figure 6:**
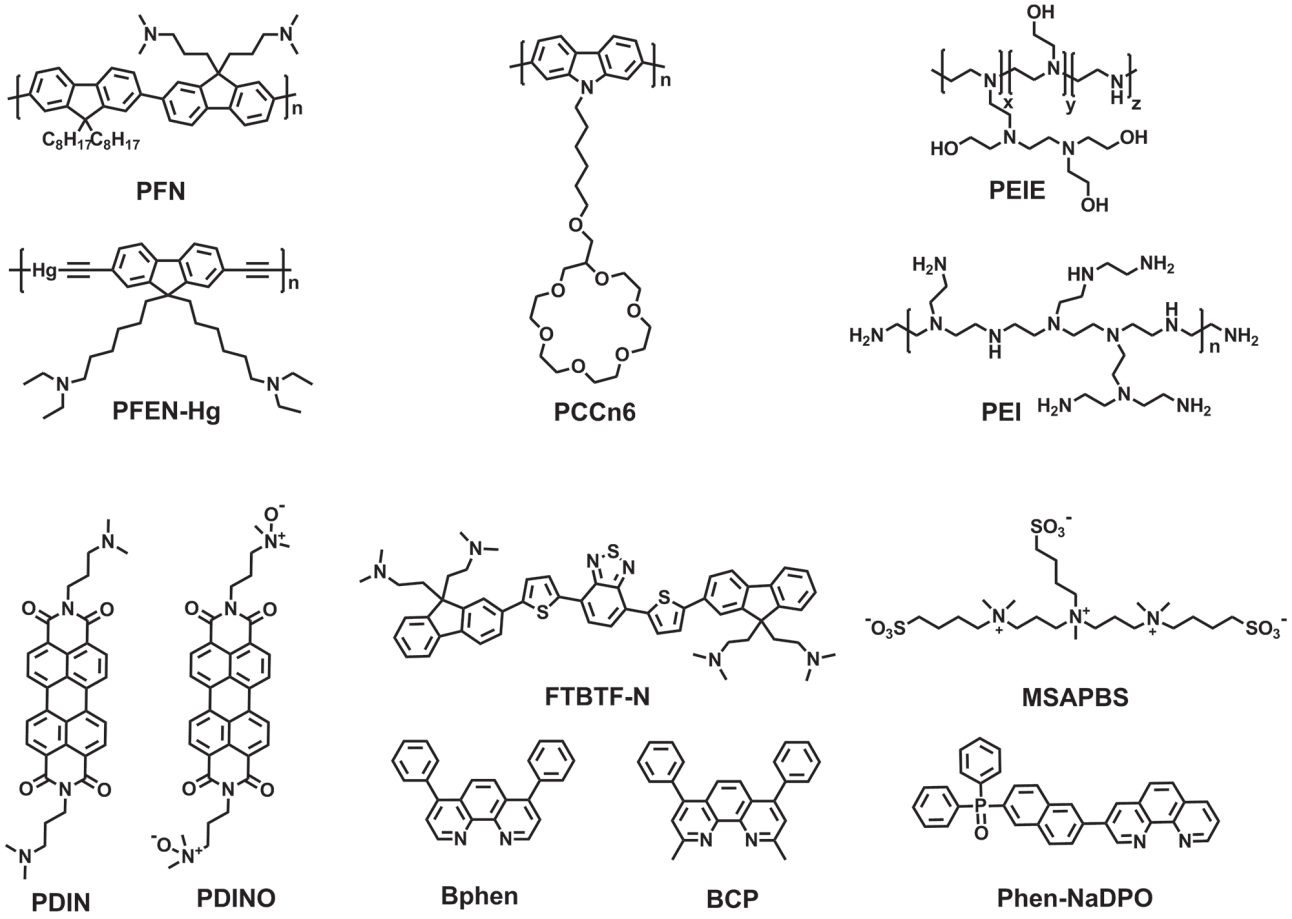
Molecular structures of some representative polymers and small‐molecules for CILs.

In recent years, to avoid thermal annealing of interlayers and improve their compatibility with organic active layer, solution‐processable polymers and small‐molecules are often used as interface materials for improving solar cell performance. The structures of organic molecules can be easily modified towards suitable energy levels and optical/electronic properties. Owing to the intermolecular dipole moment and the ability to form self‐assembled monolayers, organic CILs can induce an interface dipole pointing from the cathode to the active layer in the device geometry, thereby effectively reducing the WF of cathodes and increasing the built‐in potential of OSCs.^36^ It should be mentioned that the progress of polymer CILs for both conventional and inverted OSCs has been discussed in a recent review.^116^ Thus, we will only discuss some representative and the latest examples of polymers/small‐molecules as CILs for OPV applications in this subsection. The molecular structures of the polymers and small‐molecules used in this section are shown in **Figure** **6**.

Water/alcohol soluble conjugated polymers are effective CILs for OSC applications. In this type of materials, the π‐conjugated main chains render them delocalized electronic structures, while the polar pendant groups on their side chains can improve their solubility in water and polar organic solvents. These materials can efficiently adjust the WF of cathodes by forming an interfacial dipole between the cathode and the active layer, benefiting the charge transport. Due to their ambient solution processibility, a lot of water/alcohol soluble conjugated polymers were designed as CILs for efficient electron injection/transport in OSCs.^117^, ^118^, ^119^, ^120^, ^121^ A representative example is poly[(9,9‐bis(3′‐(*N*,*N*‐dimethylamino)propyl)‐2,7‐fluorene)‐*alt*‐2,7‐(9,9‐dioctylfluorene)] (PFN in Figure 6) that has been used as a thin CIL to simultaneously enhance *V*_OC_, *J*_SC_, and *FF* in conventional OSCs, leading to an increase in PCE from 5.0% to 8.37% for the devices based on PTB7.^117^ The improved performance upon the use of PFN were ascribed to multiple functionalities of the CIL, including enhanced built‐in potential across the device due to the existed interface dipole, improved electron‐transporting properties, elimination of the buildup of space charge, and reduced recombination loss due to the increase in built‐in field and charge carrier mobility. On this basis, the same research group employed the PFN CILs to reduce the WF of ITO, offering good Ohmic contacts for photo­generated charge‐carrier collection and allowing optimum photon harvest in the inverted OSCs.^12^, ^24^ As a consequence, greatly improved PCEs as high as 9.21% and 10.61% were demonstrated for PTB7‐ and PTB7‐Th‐based devices, respectively. The inverted OSCs exhibited superior device performance and better stability with respect to the conventional OSCs, further demonstrating the effectiveness of the PFN CILs for inverted OSCs. In a series of alcohol‐soluble conjugated polymers as CILs in OSCs, the polymer (PCCn6 in Figure 6) with electron‐rich bridge nitrogen atom in the main chain exhibited the best performance in PCE promotion.^120^ The PCCn6 CIL can induce higher interfacial dipoles for improved *V*_OC_ and promote absorption of the active layer via its optical interference effect for redistribution of the optical electric field. These benefits led to a notable improvement in PCE from 5.2% to 8.13% for the device based on PTB7:PC_71_BM.

As an alternative to conjugated polymer CILs, non‐conjugated polyelectrolytes (NCPEs) with charged ionic groups in their structures can also be employed to tune WFs of cathodes so as to reduce interfacial energy barriers and increase the built‐in potential of inverted devices.^122^, ^123^ Representative NCPE interlayer materials such as polyethyleneimine (PEI), polyallylamine (PAA), and ethoxylated polyethyleneimine (PEIE) have been directly used as CILs for OSCs.^122^, ^123^, ^124^ The use of solution‐processed PEI layer can drastically reduce the WF of ITO from 4.8 to 4.0 eV, which is originated from the strong electrostatic self‐assembled dipoles created by the presence of protonated amines within the ITO/PEI cathode. As a result, the UV‐independent inverted OSCs with a PCE of 6.3% was achieved, much higher than those using other CILs such as TiO*_x_* and poly(ethyleneoxide) (PEO).

It should be noted that most efficient inverted OSCs reported so far are obtained from an ultrathin non‐conjugated/conjugated polymer CIL (such as 2–10 nm of PEIE or PFN) due to their inherently insulating nature or low conductivities. Thereby, the development of thick polymer CILs with high electrical conductivity is critical for their uses in OSCs. For example, a Hg‐containing derivative of amino‐functionalized polymer (PFEN‐Hg in Figure 6) was designed as a new electron transport layer material, which is capable of achieving good performance under a wide range of thickness (7–37 nm), due to the Hg–Hg interactions and improved π−π stacking.^125^ The PFEN‐Hg CIL showed excellent properties of film formation, effective WF adjustment, low optical absorption and good electron collection, which allowed it to significantly improve the PCEs of inverted PTB7:PC_71_BM devices from 3.18% to 9.11%. Note that this CIL material suffers from the toxicity of Hg, which limits its practical application.

In addition to the polymeric CILs, small‐molecule CILs can also be used for the OPV application due to their attractive characteristics such as well‐defined chemical structures and ease synthesis with high purity. Many thermally deposited or water/alcohol‐processable small‐molecules, such as bathocuproine (BCP in Figure 6),^126^, ^127^ bathophenanthroline (Bphen in Figure 6),^128^ pyridinium salt‐based molecules,^129^ triazine‐ and pyridinium‐based small molecules,^130^ zwitterions,^131^ amino acids or peptides,^132^, ^133^ and self‐assembled ionic liquid (IL),^134^ have been reported as effective CILs for OSCs. Based on these studies, it can be found that small‐molecule CILs with deep HOMO levels, low WFs, good morphology stability are favorable for improving the PCE and stability of conventional/inverted OSCs. The performance enhancement by these CILs is attributed to their increased electron selectivity, better energy‐level matching for efficient charge‐transfer, and improved Ohmic contact between the cathode and the organic active layer.

For instance, Cnops et al. recently reported the use of BCP as an electron‐injection and exciton‐blocking CIL to achieve high performance fullerene‐free OSCs, which were fabricated by sequentially depositing two acceptor layers of boron subnaphthalocyanine chloride (SubNc) and boronsubphthalocyanine chloride (SubPc) on top of an α‐sexithiophene (α‐6T) donor layer.^135^ The energy‐relay cascade in the conventional device enabled an efficient two‐step exciton dissociation process, leading to a record high PCE of 8.4% for the thermally deposited single‐junction OSCs. Alternatively, two alcohol soluble small‐molecules, with perylene diimides as the core and amino (PDIN in Figure 6) or amino N‐oxide (PDINO in Figure 6) as the terminal substituent, were reported for conventional OSCs by Zhang and co‐workers.^136^ The high conductivities, appropriate energy levels and effective WF tuning in the two CILs made the conventional device work well with a wide range of CIL thicknesses. Specifically, the resulting devices (PBT7‐Th:PC_71_BM) with the PDINO CIL showed a PCE of 8.36%, much higher than that of the Ca/Al device (7.52%). Based on triarylphosphine oxide with a 1,10‐phenanthrolinyl unit, a small molecule CIL of Phen‐NaDPO (Figure 6) designed by Tan et al. exhibited a good electron mobility of 3.3 × 10^−4^ cm^2^ V^−1^ s^−1^ comparable to that of BPhen.^137^ Benefiting from the WF reduction of electrodes and the facilitation of electron extraction in OSCs, a PCE of 8.56% was obtained for the PTB7:PC_71_BM‐based device with the thermally deposited Phen‐NaDPO CIL/Al cathode, which is much higher than that of the reference Al and Ca/Al devices. The synthesis of an alcohol‐soluble molecule (FTBTF‐N in Figure 6) with two fluorene units linked by bisthiophenyl‐benzo[*c*][1,2,5]thiadiazole was reported. When FTBTF‐N was used as a CIL for inverted PTB7:PC_71_BM‐based OSCs, a high PCE of 9.22% was achieved.^138^ It was found that the introduction of two thiophene units to the backbone of the small molecule CIL materials favored more hydrophobic properties of FTBTF‐N, leading to the formation of better active layer morphology and subsequently improved *J*_SC_ and *FF* values.

Very recently, Ge and co‐workers designed a small molecule electrolyte without conjugated moieties for conventional OSCs with PCEs over 10%.^139^ The solution‐processed 4,4′‐(((methyl(4‐sulphonatobutyl)ammonio)bis(propane‐3,1‐diyl))bis(dimethyl‐ammoniumdiyl)) bis‐(butane‐1‐sulphonate) (MSAPBS in Figure 6) film, was adopted as a CIL to modify the Al cathode in conventional PTB7:PC_71_BM devices. This MSAPBS material has a deep LUMO level of −2.31 eV and a high vertical electron mobility of 1.18 × 10^−4^ cm^2^ V^−1^ s^−1^ (8 nm thickness), which can provide good contact for photogenerated charge carrier collection and allow optimum photon harvesting in the conventional OSCs. Compared to the devices with PFN or Ca CILs, a greatly improved *J*_SC_ of 19.37 mA cm^−2^ for the conventional device using the MSAPBS CIL was achieved, finally leading to a high PCE of 10.02%. This new finding using non‐conjugated small molecule CILs for OSCs has good potential in improving the performance of other optoelectronic devices.

The significant advances in polymers/small‐molecules for interface materials demonstrate that they are very useful for high performance OSCs due to their flexible structures, facile solution processability, and good interfacial compatibility with organic active layers. Nevertheless, it is noteworthy that the organic CIL materials are always sensitive to their film thickness because of their intrinsically low conductivities. The successful design of organic CILs is challenging because the ideal CILs require a universal material that combines high conductivity, good tunability of WF, appropriate film morphology, and orthogonal solvent processability.

### 2.3 Low WF Metal and Metal Salts/Complexes

Besides the mostly used metal oxides and organic materials for CILs, low WF metals and metal salts/complexes have also been developed as electron‐transport materials for OSCs. Low WF metals such as Ca, Mg, Ba, and Al were incorporated as CILs to reduce WFs of cathodes and to make Ohmic contacts with fullerene for efficient electron extraction, subsequently enhancing the photovoltaic performance of OSCs.^140^, ^141^, ^142^ By using ultrathin metals to modify ITO, inverted OSCs based on the CIL with a lower WF metal such as Ca (2.9 eV) and Mg (3.6 eV), showed much higher *V*_OC_s and PCEs than those with a higher WF metal such as Al (4.3 eV) and Ag (4.7 eV).^140^, ^141^ The inverted OSCs using Ca and Mg CILs, also achieved much better air stability compared to their conventional devices. A thin Ba CIL was inserted between the Al and organic active layer to improve PCEs of conventional OSCs based on a small molecule donor, 7,7′‐(4,4‐bis(2‐ethylhexyl)‐4H‐silolo[3,2‐b:4,5‐b′]­dithiophene‐2,6‐diyl)bis(6‐fluoro‐4‐(5′‐hexyl‐[2,2′‐bithiophen]‐5‐yl)­benzo[c][1,2,5] thiadiazole) (p‐DTS(FBTTh_2_)_2_) and the PC_71_BM acceptor.^142^ The Ba CIL can prevent trap assisted Shockley‐Read‐Hall recombination at the interface, and with different Ba thicknesses the recombination shifted from monomolecular to bimolecular. An increase in the built‐in potential after the insertion of a low WF Ba CIL further assisted faster sweep‐out. As results, the *FF* and PCE increased from 56% to 75.1% and from 5.86% to 8.57%, respectively. However, it should be mentioned that the low WF metal interlays, such as Ca and Ba, are very sensitive to moisture and oxygen that always causes the formation of detrimental quenching sites at areas near the interface between the active layer and cathode. And the low WF metal CILs need to be prepared by vacuum deposition, which limits their practical applications in low‐cost and large‐area fabrication of OSCs.

Compared to low WF metal interlayers, solution‐processable metal salts greatly broaden the available choices of CIL materials. As a substitution for commonly used metal salt of LiF, versatile metal salts such as alkali carbonates (e.g., Cs_2_CO_3_, Li_2_CO_3_, Na_2_CO_3_, etc.),^143^, ^144^ cesium acetate,^145^ cesium or sodium halides (i.e., CsI, CsCl, CsF, NaI),^146^, ^147^ cesium stearate (CsSt),^148^ and disodium edentate^149^ have been designed as CILs for OPV applications. These materials exhibited facile solution processability and low WFs, which favor the formation of interfacial dipoles between the active layer and electrode, and increase the photogenerated charge collection.^146^, ^148^ The incorporation of metal salt CILs in OSCs can also improve the wettability between the electrode and the hydrophobic organic active layer surface, resulting in better interfacial contacts and reduced contact resistances.^145^ All these benefits contribute to the PCE enhancement in devices. Nonetheless, the disadvantage of this type of CILs is that the metal salts are easy to decompose and further to diffuse into the organic layer or electrodes,^147^ which could induce a device degradation.

Additionally, several classes of metal complexes, including copper chelates,^150^, ^151^ zinc chelates,^152^ titanium chelates,^153^ and zirconium chelates^154^, ^155^ were used to improve the efficiency and/or stability of OSCs. These metal complex CILs can be made by thermal‐deposition or solution processing from protonic solvents. Their relatively high vertical conductivity and electron mobility, as well as suitable energy levels render them to reduce the WF of cathodes, decrease contact resistance, and facilitate charge collection in devices.^150^, ^151^, ^152^, ^153^ A good example is the zirconium acetylacetonate (ZrAcac) material that was designed as a universal CIL for various BHJs with enhanced performance.^154^ This CIL can be facilely prepared by spin‐coating its ethanol solution on active layers without any other post‐treatments. When adopting a low‐bandgap polymer, PBDTBDD (poly(((4,8‐bis(5‐(2‐ethylhexyl)thiophen‐2‐yl)­benzo[1,2‐b:4,5‐b′]dithiophene‐2,6‐diyl)bis(trimethyl))‐*co*‐(5,7‐bis(2‐ethylhexyl)benzo[1,2‐c:4,5‐c′]dithiophene‐4,8‐dione))), as the donor and PCBM as the acceptor, a best PCE of 9.23% was achieved for the conventional OSCs with ZrAcac CILs, which was much higher than that of the devices with Al (5.72%) and traditional Ca/Al (7.34%) as cathodes. The high performance can be attributed to the decreased series resistance, enhanced electron‐extraction and light harvesting of the devices using the ZrAcac CIL. Compared to a 56% decay in PCE for the OSCs with the Ca interlayer, the improved stability with a small PCE decay (≈7%) was demonstrated for the conventional device with the stable ZrAcac CIL under 30 days storage in N_2._

### 2.4 Carbon‐Based Materials

Carbonaceous materials are considered as a promising class of candidates for CILs with their high conductivity, good structural stability and tunable functionality. To date, fullerene, carbon nanotube (CNT), and graphene as well as their derivatives have been developed as effective CIL components.^21^, ^156^, ^157^, ^158^

**Figure 7:**
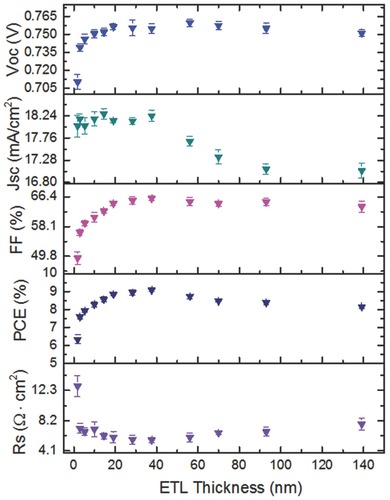
Thickness dependence of device performance on the C_60_‐SB CIL (error represents ±1 standard deviation over eight devices). Reproduced with permission.^164^

Among the carbon‐based CILs, fullerene‐derived materials have unique virtues of being structurally similar to the fullerene acceptors, which can smoothly bridge electrons extracting/transporting from the fullerene acceptor to the cathode.^118^, ^159^, ^160^ A solution‐processable amine group functionalized fullerene derivative, DMAPA‐C_60_, was designed as a versatile CIL for enhancing the performance of different polymer/fullerene devices.^[161a]^ Using this CIL to fabricate small‐molecule conventional OSCs, the efficiency improvement was observed in contrast with devices using ZnO, Ca, or Ba as the CIL due to the improved interfacial compatibility and reduced recombination at the interfaces.^[161b]^ More importantly, the good outdoor stability was found for the conventional OSCs using the stable DMAPA‐C_60_ CIL, which maintained ≈90% of their initial PCEs after 30 days storage without any encapsulation. Note that a trade‐off between the oxidative stability and WF of metal electrodes is a major challenge. To solve this problem, tris(dimethylamino)–substituted fullerene (C_60_‐N) and tris(sulfobetaine)‐substituted fullerene (C_60_‐SB) were designed as cathode‐independent CIL materials for conventional OSCs.^160^ The optimized devices (PTB7‐Th:PC_71_BM) with C_60_‐N or C_60_‐SB CILs yielded average PCEs of 9.35% and 8.57%, respectively. Particularly, a thin C_60_‐N CIL reduced the WFs of Ag, Cu, and Au electrodes to 3.65 eV. As a result, PCEs exceeding 8.5% were obtained for OSCs independent of the cathode used (Al, Ag, Cu, or Au). Meanwhile, combining ideal energy levels, and excellent electron‐transporting ability, hydrophilic fullerene derivatives can be a promising class of CIL materials for efficient inverted OSCs.^162^, ^163^, ^164^ For example, C_60_‐SB has dual functionalities of acting as a thickness insensitive CIL and as an electron acceptor in inverted OSCs.^164^ Average PCEs as high as 9.08% (a maximum PCE of 9.23%) were obtained for the inverted PTB7‐Th:PC_71_BM‐based OSCs with a ≈40 nm C_60_‐SB CIL. Excellent insensitivity to the CIL thickness of 5 to 140 nm was found, and PCEs over 8% across the entire thickness range (**Figure** **7**) were achieved for the OSCs. Another good example is the design of a fullerene‐based CIL made from a stable conductive fulleropyrrolidinium salt embedded in a thermally cross‐linkable fullerene matrix.^162^ When used for inverted OSCs, the in situ doping and cross‐linking improved both the conductivity and solvent resistance of the CILs. Thereby the significantly improved PCEs from 2.43 to 5.26% were achieved.

Graphene and its derivatives have a unique 2D structure, high transparency, and their WFs can be tuned through simple chemical modifications. Therefore, they have also been developed as a new type of interlayers for OSCs.^158^ For instance, solution‐processed graphene quantum dots were adopted as CILs to facilitate charge transfer and reduce charge recombination in inverted OSCs due to their low WFs and unique optical/electronic properties.^165^, ^166^ The use of –COOCs groups to replace periphery –COOH groups in graphene oxide (GO) generated a good electron‐transporting cesium‐neutralized GO (GO‐Cs) layer.^167^ This GO‐Cs CIL modified cathode afforded a low WF of 4.0 eV, matching the LUMO level of PCBM for efficient electron‐extraction. Consequently, conventional and inverted OSCs with the GO–Cs CILs exhibited comparable performance to that of the standard OSCs using the state‐of‐the‐art CIL materials. To achieve efficient electron transport in OSCs, a transferable GO layer with a WF of 4.3 eV was fabricated by graphene stamping and subsequent oxidation with HNO_3_.^168^ This GO CIL can reduce the series resistance and improve the *J*_SC_ because of the efficient electron extraction/transport from the BHJ (PCDTBT:PC_71_BM) to the Al cathode. As a consequence, the GO CIL‐based OSC achieved a PCE of 6.72%, comparable to that of the TiO*_x_* CIL‐based OSC. With a sequentially deposited GO and TiO*_x_* as the CIL, a further improved PCE of 7.5% was obtained. Furthermore, the device using the GO CIL exhibited a much higher stability (a 3% PCE decay) in comparison with the device without the GO interlayer (a 56% PCE decay), indicating their beneficial supporting for enhanced device lifetime. When PCBM was attached to reduced graphene oxide (rGO) via noncovalent π–π interaction, a new graphene‐fullerene composite (rGO‐pyrene‐PCBM) CIL was designed for efficient P3HT:PCBM OSCs (PCE = 3.89%).^169^ By contrast, the OSCs using the rGO (pyrene‐PCBM) component as a CIL showed dramatically decreased PCEs of 2.53% (2.18%), suggesting the importance of composite formation between rGO and pyrene‐PCBM components for its electron extraction property.

### 2.5 Inorganic–Organic Hybrids and Composites

A variety of electron‐transporting materials developed in recent years can provide ample building blocks to further design high performance CIL materials for OSCs. The concept of designing hybrid/composite CILs is well recognized because high performance OSCs have been achieved in such new architectures.^25^, ^170^ By combining the advantages of inorganic, organic and other useful components, the hybrid/composite electron‐transporting CIL materials are excellent candidates to engineer the interfaces of OSCs. Therefore, there are growing interests to develop hybrid/composite CIL materials toward OSCs with high efficiency and good stability.

Among various hybrid/composite CIL materials, organic‐inorganic CIL materials are a major and very significant family of electron‐transporting materials for OSC applications. Lots of organic materials such as polymers and small‐molecules can be used to dope with inorganic metal oxides or other inorganic compounds, functioning as hybrid/composite CILs. A large number of solution‐processed hybrid CILs such as ZnO‐poly(vinylpyrrolidone),^170^ PEO‐modified ZnO,^171^ ZnO‐PFN and ZnO‐diethanolamine,^172^, ^173^ TiO_2_:1,10‐phenanthroline^174^ and Cs_2_CO_3_‐doped BPhen,^175^ etc. have been developed to enhance device efficiency and stability. These hybrid CIL materials can combine merits of both inorganic and organic parts, thus providing a controllable way to circumvent the inherent weaknesses of each component, and tune the interface compatibility and characteristics. The tailored WFs of these hybrid/composite CILs can greatly enhance electron‐transport and reduce charge recombination within devices. All of these features are greatly beneficial for high performance OSCs. Although inorganic or organic CILs also have decent device performance, organic‐inorganic hybrid CIL materials may have better interface compatibility with the active layer and the cathode.

**Figure 8:**
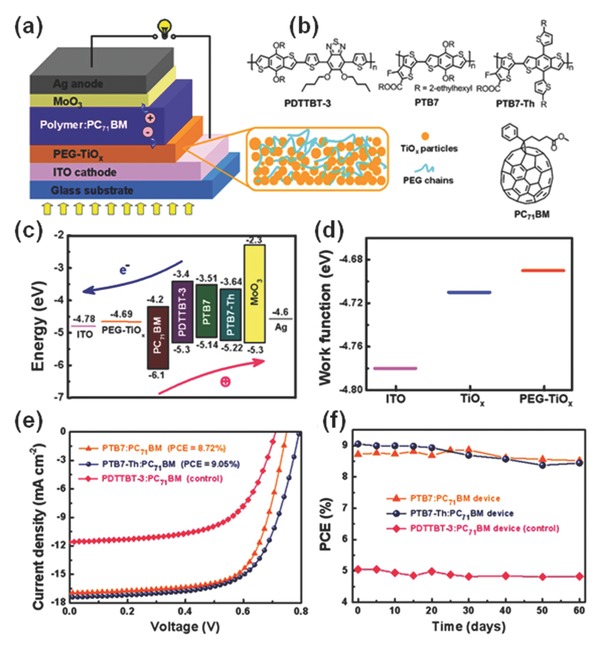
a) Schematic illustration of the inverted OSC with a hybrid PEG–TiO*_x_* CIL. b) Molecule structures of PDTTBT‐3, PTB7, PTB7‐Th and PC_71_BM. c) Energy levels of each component used in the OSCs. d) WFs of ITO, TiO*_x_* and PEG–TiO*_x_* films determined by a Kelvin probe technique. Improved device performance of different polymer‐based OSCs using the PEG–TiO*_x_* CIL: e) *J–V* characteristics under AM 1.5G irradiation (100 mW cm^−2^), and f) Device stability of the OSCs. Reproduced with permission.^27^ Copyright 2015, Springer.

Recently, by hybridizing poly(ethylene glycol) (PEG) with sol–gel TiO*_x_*, a universal electron‐transporting CIL material was designed for OSCs based on different polymer blends, where the corresponding device structure, components and energy levels are shown in **Figure** **8**a–c.^27^ The hybrid PEG–TiO*_x_* CIL offered advantages of facile solution processability, low annealing temperature (150 °C), low‐cost and safety with cheap and environment‐friendly raw materials, good interfacial/optoelectronic properties. Similar to TiO*_x_*, when the hybrid PEG–TiO*_x_* was used to modify ITO, a reduced WF was found (shown in Figure 8d). The reduced WF of PEG–TiO*_x_* might be ascribed to the fill‐up of TiO*_x_* surface traps by the lone pair electron of oxygen atoms in the PEG backbone, and the surface dipole moments pointed outwards from the ITO. Therefore, this hybrid PEG–TiO*_x_* CIL can facilitate electron injection/transport by reducing energy barriers between the BHJ layer and ITO, which can be used as a novel class of CILs for improving electron‐collection and reducing interface energy barriers in various BHJ systems. Device performance based on this hybrid CIL was found to be much better than that with the pure TiO*_x_*. With the PEG–TiO*_x_* CIL, inverted OSCs delivered greatly improved PCEs up to 9.05% (Figure 8e), representing a record high efficiency for inverted OSCs with TiO*_x_*‐based interlayers. More importantly, using this PEG‐TiO*_x_* CIL, these high efficiency OSCs were verified to have good stability (Figure 8f). The best devices of the PTB7 and PTB7‐Th system maintained their high performance over two months of storage (PCEs of 8.52% and 8.44%, respectively). Accordingly, both improved PCEs and stability were demonstrated for the different polymers‐based devices with the hybrid PEG–TiO*_x_* CIL, demonstrating its universality for enhancing device performance of OSCs. The use of hybrid PEG–TiO*_x_* composites could also be extended to other solar cell systems considering their excellent optoelectronic and interfacial properties.

By adopting a ferroelectric polymer, poly­(vinylidenefluoride‐*co*‐trifluoroethylene) (P(VDF‐TrFE)) to blend with ZnO, Cho et al. designed an *n*‐type ferroelectric blend layer (FBL) which can be employed as a hybrid CIL with non‐Ohmic contact electrodes under various electrical bias conditions.^176^ With this carrier‐selectivity‐controlled CIL, an increase in PCE from 6.93% (without poling) to 8.15% (after positive poling) was demonstrated for the inverted OSCs based on the PTB7:PC_71_BM BHJ. The noticeable increased *FF* up to 76.5% agreed well with the expectations that the internal field formation should be improved even with non‐Ohmic contact electrodes owing to the suppressed interfacial recombination by the poling of the FBL.

**Figure 9:**
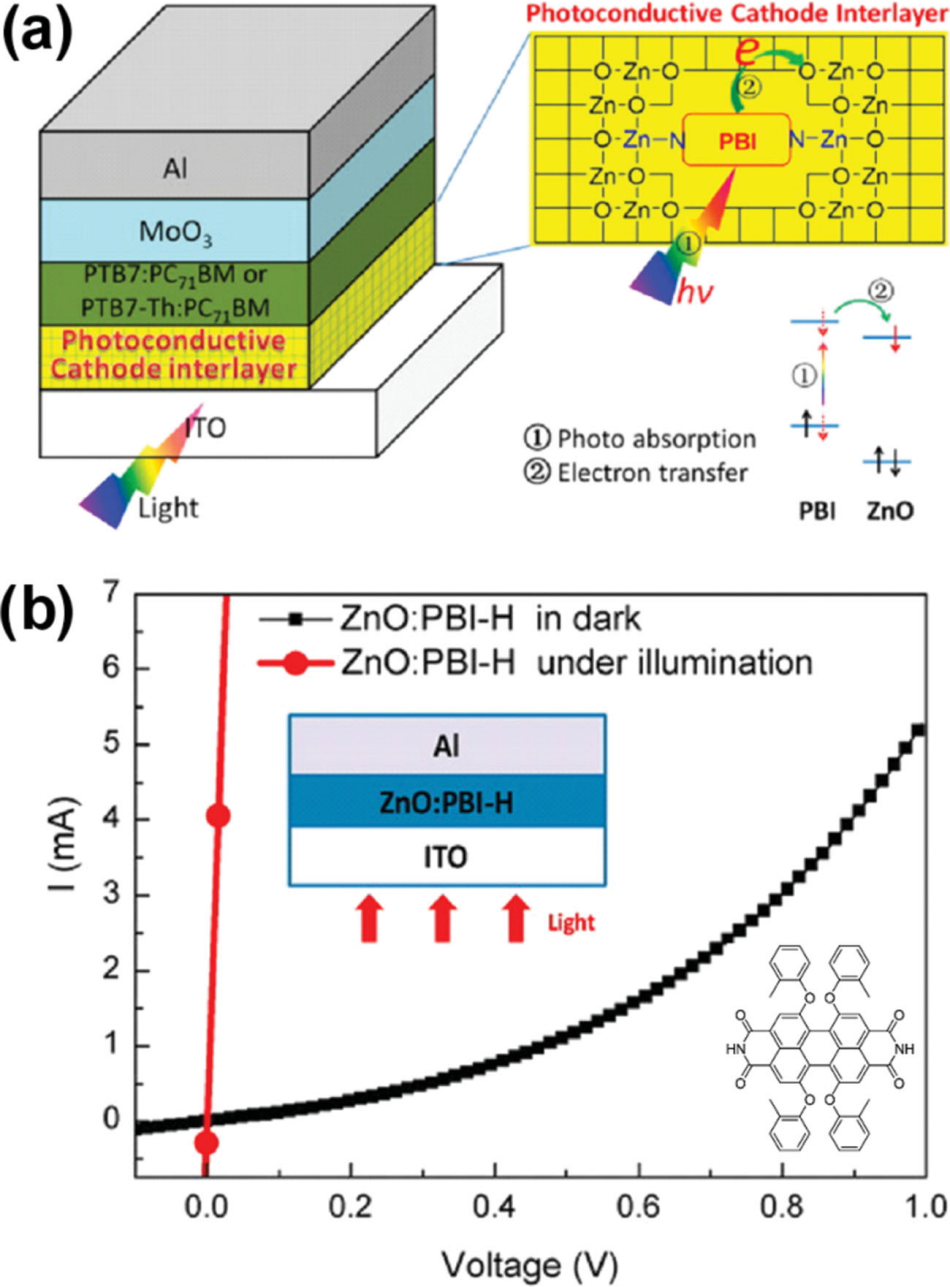
a) Schematic device structure based on the hybrid CIL of ZnO:PBI‐H. b) *I*−*V* curves for the device with ITO/ZnO:PBI‐H (90 nm)/Al in the dark and under AM 1.5G illumination. The inset is the chemical structure of PBI‐H. Reproduced with permission.^177^ Copyright 2015, American Chemical Society.

By using a dye to modify inorganic ZnO, Nian et al. reported a highly photoconductive CIL material for inverted OSCs (**Figure** **9**a).^177^ This hybrid CIL was achieved by doping a small amount of light absorber such as PBI‐H (shown in the set of Figure 9b) into a sol−gel‐derived ZnO, where the PBI‐H molecules could form a N−Zn bond with ZnO during the thermal treatment. Electron mobility of the CIL was improved from 5.10 × 10^−4^ (ZnO) to 2.02 × 10^−3^ cm^2^ V^−1^ s^−1^ (ZnO:PBI‐H). As shown in Figure 9b, the *I−V* curve transition suggests that the ZnO:PBI‐H CIL transforms from semiconductor in dark to conductor under illumination with the electrical conductivity of ≈4.50 × 10^−3^ S m^−1^. The high photoconductivity of this CIL can be understood in terms of the photoinduced electron transfer from PBI‐H to ZnO (Figure 9a), which largely enhances the electron concentration on the conduction band of ZnO. Inverted OSCs using this novel photoconductive CIL delivered greatly improved device performance, which was rather insensitive to the CIL thickness over a broad thickness range of 30−60 nm. As a result, the inverted devices based on ZnO:PBI‐H increased from 8.45% to 10.59% (7.58% to 9.09%) for the PTB7‐Th:PC_71_BM (PTB7:PC_71_BM) system. This kind of hybrid photoconductive film is very valuable for the development of high performance CIL materials, and the photodoping concept may be applicable in other organic electronic devices.

Besides directly acting as a doping component in hybrid/composite CILs, organic materials such as conjugated/nonconjugated polymers,^88^, ^178^, ^179^ small molecules,^180^, ^181^ self‐assembled monolayers (SAMs),^60^ were also implemented as an interlayer to modify metal oxides, thus forming a bilayer CIL for various OSCs. Surface/interface modification of metal oxides with these organic matters can passivate the surface traps/defects of inorganic metal oxides, reduce the WFs, suppress the recombination loss of carriers, and improve the electrical coupling of metal oxide/active layer. Consequently, considerably improved *V*_OC_, *J*_SC_ and *FF*, and final PCE values of OSCs are expected. Using a non‐conjugated PEI thin‐layer to modify ZnO as the composite CIL, the resulting PTB7:PC_71_BM‐based inverted OSCs delivered an improved PCE up to 8.88%, much better than those of devices using the pure ZnO (6.99%) or PEI (7.49%) CIL.^182^ The enhanced PCE by using this hybrid bilayer ZnO/PEI CIL was attributed to the lowered conduction band energy of the ZnO layer via the formation of an interfacial dipole at the ZnO/PEI interfaces. The noticeably decreased series resistance of devices resulting from the use of hybrid CILs was another reason for the PCE enhancement. Compared to devices with ZnO, a less decay in PCE was observed for the ZnO/PEI CIL‐based devices without encapsulation, further demonstrating the improved stability with this hybrid CIL. Similar results were also documented in solution‐processed PEIE‐modified ZnO CILs, by which inverted small‐molecule OSCs with enhanced PCEs up to 7.88% were achieved.^178^ This hybrid CIL‐based inverted OSCs were relatively stable in air compared to the conventional devices, demonstrating a promising way to the fabrication of small‐molecule OSCs with high efficiency and long‐term stability. Interface modification of ZnO with organic small‐molecules is also beneficial to enhance device stability due to the partial passivation of the metal oxide surface to molecular oxygen adsorption.^180^ Surface passivation while maintaining the WF control of a selective CIL can be utilized to improve PCE and lifetime of OPV devices. Surface energy control of ZnO by using SAMs with different polar end groups led to successful morphology control of the organic active layer, thereby improving the device performance.^60^ Moreover, when an ion‐liquid of 1‐butyl‐3‐methylimidazolium tetrafluoroborate ([BMIM]BF_4_) was used to modify ZnO, the resulting device showed increased *J_SC_* and *FF* as a result of the reduction in the WF of the cathode because the modified ZnO can form spontaneous dipolar polarization at the interface.^181^ The PCE of the device using the [BMIM]BF_4_‐modified ZnO CIL was increased to 10.15% in comparison with a PCE of 8.94% for the reference device based on the regular ZnO CIL.

In addition, carbon‐based materials can be integrated with metal oxides (or organic materials) as surface modifiers or doping components to design hybrid/composite CILs. Incorporation of PCBM into ZnO CILs can modulate the electronic and orbital interactions at the interfaces of inverted OSCs to improve their *J_SC_* and *FF*.^183^ The use of fullerene‐based SAMs was efficient in promoting charge transfer between the metal oxide and the active layer, leading to improved PCEs by passivating the surface trap sites of metal oxides and reducing the contact resistances.^21^, ^184^ Meanwhile, fullerene materials can be incorporated into metal oxides to get novel hybrid CILs, which provide dual functionalities for improved electron extraction/collection, including the formation of a fullerene‐rich CIL surface and the promotion of electron conductivity at the interface and bulk.^25^, ^26^ When used for constructing inverted OSCs (PTB7‐Th:PC_71_BM), an increased PCE from 7.64% to 9.35% for a fullerene derivative‐doped ZnO (ZnO‐C_60_) CIL was demonstrated by Liao and co‐workers.^25^ With the similar method, a hybrid CIL of fullerene and ternary IZO was developed, leading to an improved PCE as high as 10.31% in inverted OSCs.^26^ The fullerene derivatives such as fulleropyrrolidinium iodide (FPI), were blended with PEIE to improve their conductivity and WF tunability of the hybrid FPI‐PEIE CILs.^185^, ^186^ Using solution‐processed FPI‐PEIE as CILs, high PCEs up to 9.62% were achieved in inverted OSCs based on poly[4,8‐bis(5‐(2‐ethylhexyl)thiophen‐2‐yl)benzo[1,2‐*b*;4,5‐*b*′]dithiophene‐2,6‐diyl‐*alt*‐(4‐(2‐ ethylhexyl)‐3‐fluorothieno[3,4‐*b*]thiophene)‐2‐carboxylate‐2,6‐diyl] (PBDTT‐TT):PC_71_BM.^185^ Adopting this highly conductive CIL, an ITO‐free flexible OSC with a high PCE of 10.4% was achieved through the innovative design and integration of ultrathin metal film electrode and efficient BHJ into a microcavity‐based architecture.^186^ Similarly, 2D rGO sheets were attached to ZnO or TiO*_x_* as composite CILs for enhancing solar cell performance. Inserting an rGO‐TiO_2_ CIL between the active layer and Al cathode, the conventional OSCs showed an improved PCE from 4.15% to 5.33%.^187^ The enhanced performance is mainly benefited from the dual role of rGO‐TiO_2_ as an optical spacer and an electron‐extracting layer. The design of rGO‐modified ZnO or TiO*_x_* composites can achieve a fast electron transfer path in the CIL, reduce charge recombination at organic/inorganic interfaces, and weaken the sensitivity of CILs to environment, finally increasing PCEs and stability of the inverted OSCs.^188^, ^189^

### 2.6 Other Emerging Alternatives

Although many electron‐transporting materials have been demonstrated as CILs in the previous several subsections, some unique CIL materials for OSCs are still not included, and thus they will be discussed in this subsection.

**Figure 10:**
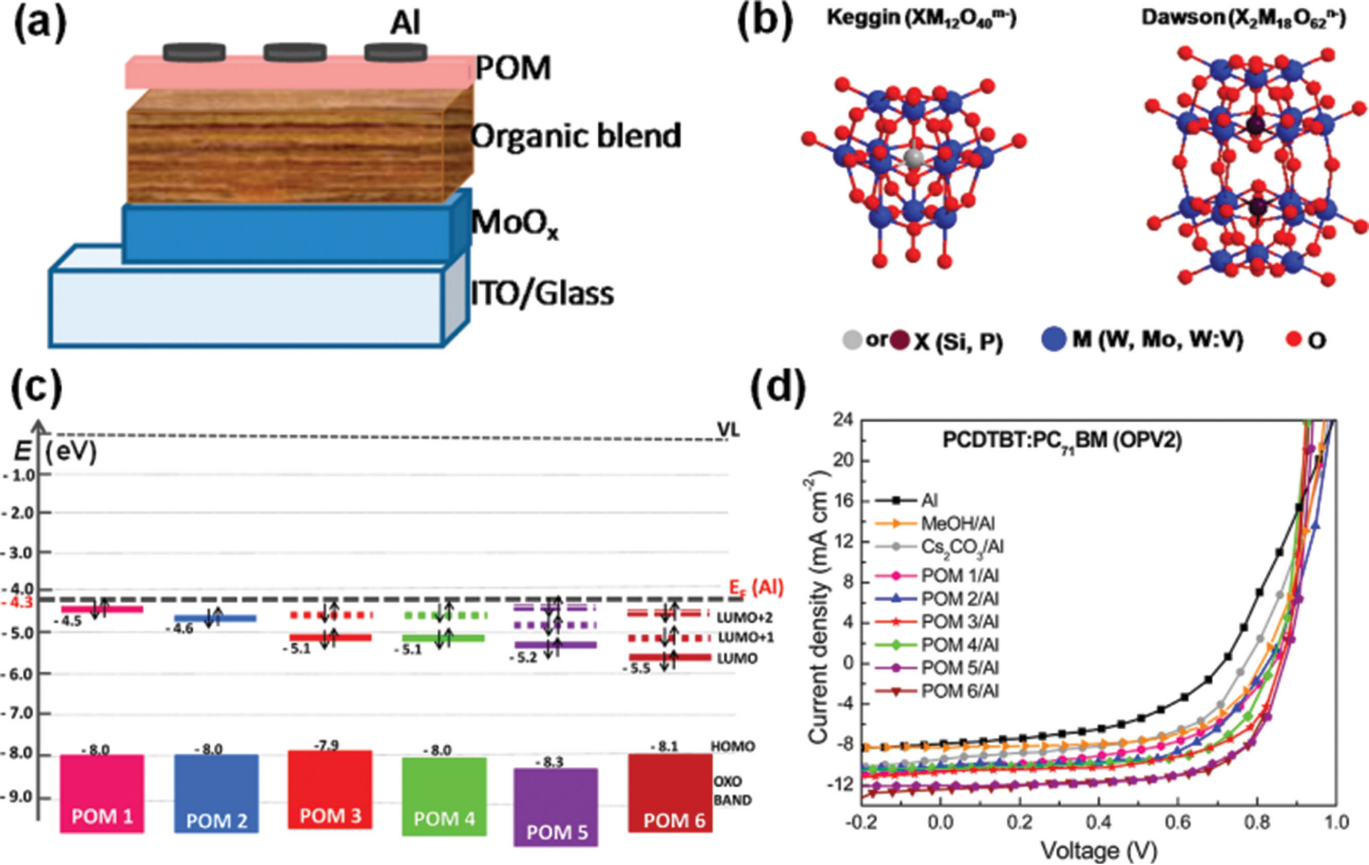
a) A schematic conventional OSC based on the polyoxometalate (POM) CIL. b) Molecular structures of Keggin and Dawson POMs used in this study. c) Molecular orbital diagrams of various POMs. d) *J–V* characteristics under AM 1.5G illumination of the OSCs using various CILs. Reproduced with permission.^190^ Copyright 2015, American Chemical Society.

An interesting example is polyoxometalates (POMs) that have been developed as a new class of electron‐transporting materials in organic electronics.^190^ Because POMs are a well‐known group of clusters with cage like structures built from transition metal oxo anions linked by shared oxide ions, they can be spin‐coated from a methanol solution onto the organic active layer for serving as CILs in conventional OSCs (**Figure** **10**a). The typical structures of the POM clusters as CILs are shown in Figure 10b. Among them, the Keggin POMs are H_4_SiW_12_O_40_ (POM 1), H_3_PW_12_O_40_ (POM 2), H_5_PV_2_W_10_O_40_ (POM 3) and H_3_PMo_12_O_40_ (POM 4), while the Dawson POMs are (NH_4_)_6_P_2_W_18_O_62_ (POM 5) and (NH_4_)_6_P_2_Mo_18_O_62_ (POM 6). Energy levels of all POMs are compared with the Fermi level of Al (4.3 eV) in Figure 10c. The substitution of the metal centers in a Keggin structure with other more electronegative ones or/and transition to a Dawson structure leads to a higher degree of reduction because of the decreased LUMO energy. The Keggin and Dawson POMs can be reduced at their interfaces with Al cathode, and the degree of reduction was strongly influenced by the position of their LUMO levels. In the OSCs, the reduced POM CILs contributed to the formation of an Ohmic contact thus facilitating electron injection/transport and the suppression of recombination losses. Depended on the degree of their reduction, device performance improvement was thus achieved for all the POM CILs. With a POM 6 CIL, conventional PCDTBT:PC_71_BM OSCs showed an improved PCE of 7.4%, among the best of these POM CILs (Figure 10d). These results indicate the good potential of POMs for promising CIL materials.

Recently, researchers also reported other emerging alternative materials as electron‐transporting CILs for OPV applications, such as n‐type metal sulfides/selenides^191^, ^192^ and several new composites/hybrids including Al_2_O_3_:ZnO,^193^ ZnO:SrTiO_3_,^194^ metal salt‐doped ZnO,^195^ rare earth nanoparticle‐doped TiO_2_,^196^ TiO_2_‐coated CNTs,^156^ Ag‐doped BCP,^197^ and so forth. All these examples largely enrich the scope of electron‐transporting materials used for CILs, and will further promote the rapid improvement of solar cell efficiency and stability in the future.

## 3 Hole‐Transporting Materials as AILs

While the electron‐transporting CILs have low WFs for electron collection, the hole‐transporting materials as AILs should have high WFs to match the HOMO levels of the donor materials in the active layer to facilitate hole‐extraction. Meanwhile, the AILs should efficiently transport holes to minimize series resistances of OSCs for good photovoltaic performance. To date, many hole‐transporting AIL materials such as organic conductive polymers, inorganic metal oxides/sulfides, graphene oxides, their hybrids/composites and other alternatives have been designed for both conventional and inverted OSCs. The device characteristics of some representative conventional or inverted devices employing various AILs are summarized in **Table** **2**.

**Table 2:** Device characteristics of some representative OSCs with different AILs

OSC type	Anode configuration	Active layer	Cathode configuration	*V*_OC_ (V)	*J*_SC_ (mA cm^−2^)	*FF* (%)	PCE (%)	Ref.
Con.	ITO/PEDOT:PSS+Ag NPs	PIDTT‐DFBT:PC_71_BM	Bis‐C_60_ +Ag NPs/Ag	0.96	14.36	63	9.02	203
	ITO/PEDOT:PSS+Ag@Au	PTB7:PC_71_BM	TiO*_x_*/Al	0.75	17.50	70	9.19	46
	ITO/PEDOT:PSS/DIP	SubNc/Cl_6_‐SubPc‐Cl	BCP:C_60_/Ag	1.04	10.1	66.6	6.86	214
	ITO/s‐MoO_*x*_	P3HT:IC_70_BA	Ca/Al	0.84	11.09	70.5	6.57	226
	ITO/NiO*_x_*	PTB7‐Th:PC_71_BM	Ca/Al	0.79	18.32	63.1	9.16	240
	Ormoclear/Ag/WO_3_	PTB7:PC_71_BM	Ca/Al	0.75	15.2	66.6	7.63	247
	ITO/ReO*_x_*	PBDTTT‐C‐T:PC_71_BM	Ca/Al	0.76	17.74	61.6	8.30	248
	ITO/RuO_2_	PBDTBDD:PCBM	Ca/Al	0.84	12.66	70.1	7.45	245
	FTO/MoS_2_/MoO_3_	P3HT:PCBM	Al	0.63	9.90	67.1	4.15	252
	ITO/Cl‐GO	PBDTTT‐C:PC_71_BM	Ca/Al	0.72	17.0	62	7.59	258
	ITO/n‐G/Au NDs	PTB7:PC_71_BM:n‐MWCNTs	PFN/Al	0.74	19.10	65.8	9.29	261
	ITO/p‐PFP‐HD	PTB7‐Th:PC_71_BM	PFN/Al	0.78	16.3	71	9.03	266
	ITO/CBA	PTB7:PC_71_BM	Al	0.74	16.47	70	8.48	267
	ITO/O‐NiAc	P3HT:ICBA	Ca/Al	0.88	10.55	72	6.64	272
	ITO/CuSCN	PDPP‐2T‐TT:PC_71_BM	Sm/Al	0.70	16.7	69	8.0	273
Inv.	PEDOT:PSS+Au NPs/Ag	PTB7‐F20:PC_71_BM	ITO/ZnO ripple/ALD‐ZnO	0.68	17.25	67.2	7.93	201
	PANI/Ag	PSEHTT:ICBA	ITO/TiO_2_/C_60_‐SAM	0.89	11.60	66.9	6.87	220
	PFT‐3HT/Ag	P3HT:PCBM	ITO/ZnO	0.60	11.3	64.9	4.6	217
	*p*‐doped P3HT/Ag	P3HT:ICBA	ITO/PEIE	0.80	7.7	63	3.9	222
	V_2_O_5_/Ag	P3HT:PCBM	FTO/TiO_2_	0.56	10.69	50.5	3.09	236
	VO*_x_*/Ag	a‐PTPTBT:PC_71_BM	ITO/ZnO	0.82	11.6	53	5.0	233
	MoS_2_/Ag	PTB7:PC_71_BM	ITO/ZnO	0.72	15.90	71	8.11	253
	GO/MoO_3_/Ag	PIDTT‐DFBT‐TT:PC_71_BM	ITO/ZnO/PCBM‐COOH	0.97	11.6	64.5	7.29	254
	sGO/VO_*x*_/Ag	PTh_4_FBT:PC_71_BM	ITO/ZnO	0.76	13.2	67	6.7	255
	WC/Ag	PTB7:PC_71_BM	ITO/ZnO	0.72	15.98	70	8.04	271

Note: Con. represents the conventional OSC; Inv. represents the inverted OSC.

### 3.1 Conducting Polymers and their Composites

As a representative conducting polymer, PEDOT:PSS is widely used as a hole‐transporting material in OSCs due to its high conductivity and easy solution‐processing. At the same time, the proper WF of PEDOT:PSS (≈5.1 eV) matches well with many polymer donors to form good Ohmic contact at the anode/active layer interface.^21^ However, the acidic and moisture sensitive nature of PEDOT:PSS significantly influences the stability of devices based on it.

So far extensive studies have been carried out to improve the performance of PEDOT:PSS AILs. Incorporating metal NPs into the PEDOT:PSS film is an effective way to optimize the corresponding performance of OPV devices, which may be mainly ascribed to the localized surface plasmon resonance (LSPR) effect. With the plasmonic light scattering effect, the absorption of active layers with metal NPs may be improved.^198^ And by tuning the size, shape and surroundings, the optical properties can be controlled.^199^, ^200^

**Figure 11:**
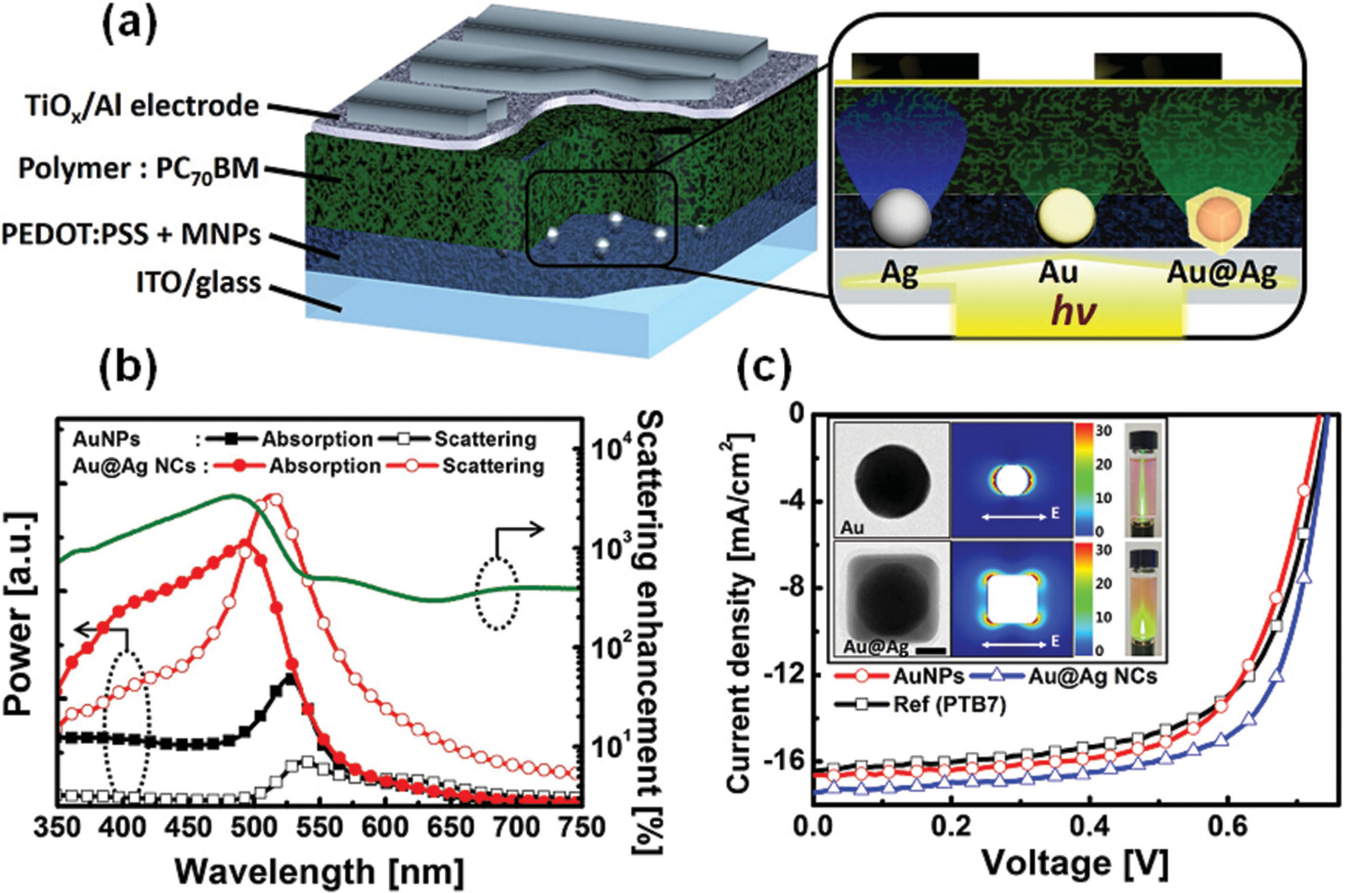
a) A schematic OSC using metal NPs‐doped PEDOT:PSS as an AIL. b) The scattering power and absorption power of the 45 nm Au NPs and 45 nm@10 nm Au@Ag NCs. The scattering enhancement of the Au@Ag NCs compared to that of the Au NPs is plotted as a green line. c) *J–V* curves of a control PEDOT:PSS device and the best plasmonic PTB7:PC_71_BM OSCs with the Au NPs and Au@Ag NCs embedded. The inset graph includes TEM images (left, scale bar: 20 nm), scattered electric field *|E|* ^2^ distributions (middle), and scattering images (right) of the Au NPs and Au@Ag NCs. Reproduced with permission.^46^ Copyright 2014, American Chemical Society.

Among many metal NPs, gold (Au) and silver (Ag) NPs have been studied extensively due to their strong LSPR properties.^201^, ^202^ By using a dual interface doping method with silver nanoprisms, enhanced optical absorption was realized, and the PCE of the conventional OSCs based on poly(indacenodithieno[3,2‐b]thiophene‐difluoro‐benzothiadiazole) (PIDTT‐DFBT):PC_71_BM increased from 7.7% to 9.0%.^203^ Through analytic optical simulations and the near‐field optical microscopy to visualize the scattering field, the size‐dependent plasmonic forwarding scattering effect of the metal NPs was verified.^46^, ^204^ As shown in **Figure** **11**, by embedding core‐shell Au@Ag nanocomposites (NCs) in PEDOT:PSS as an AIL, conventional OSCs showed enhanced plasmonic absorption and delivered an increased PCE (from 8.31% to 9.19%) in PTB7:PC_71_BM‐based devices due to the much stronger scattering efficiency of Au@Ag NCs than that of Au NPs. Similarly, to enhance the light harvesting and promote exciton dissociation and hole transport, a core‐shell structure design of silica coated Ag NPs was utilized for OSCs.^205^ Large Au@SiO_2_ core/shell NPs were also incorporated into the PEDOT:PSS AIL.^206^ It was revealed that Au@SiO_2_ NPs penetrated into BHJ layer and part of the Al electrode layer. The SiO_2_ shell can provide an insulating barrier to limit the conductivity of AuNPs and plays the role of “surfactant” fulfilling the good dispersion of AuNPs in both the PEDOT:PSS and the active layer as well. Therefore, the incorporation of NPs into BHJ‐OSC devices resulted in efficiency enhancement by ∼16%.^206^

Other modifications on PEDOT:PSS have also been carried out, such as metal salts,^207^, ^208^ polymers,^209^, ^210^ and so on.^211^, ^212^, ^213^ Recently, a high PCE of 6.9% for non‐fullerene OSCs was achieved by the insertion of a thin exciton‐blocking diindeno[1,2,3‐cd:1′,2′,3′‐lm]perylene (DIP) layer between the active layer and the anode.^214^ PEDOT:PSS provided a smooth surface for the deposition of a closed DIP layer, which contributed to the improved *J*_SC_ and the increased *V*_OC_. The large improvement on the photocurrents was also caused by the reduced exciton quenching at the anode.^215^ Interface gap optimization and the selection of buffer layers are both important for the efficient charge extraction and transportation. To take the advantage of the good stability of MoO_3_, an AIL was fabricated by in situ formation of MoO_3_ in aqueous PEDOT:PSS dispersion.^216^ The PCE of the conventional device was increased from 5.5% to 6.4%, and the stability was significantly improved too.

Conducting polymers apart from PEDOT:PSS were employed as AILs in OSCs.^217^, ^218^, ^219^ By incorporating solution‐processable polyaniline (PANI) as AILs, the flexible OSCs based on poly[(4,4′‐bis(3‐(2‐ethyl‐hexyl)dithieno[3,2‐b:3′‐d]silole)‐2,6‐diyl‐*alt*‐(2,5‐(3‐(2‐ethylhexyl)thiophen‐2‐yl)thiazolo[5,4‐d]thiazole)] (PSEHTT):ICBA exhibited a high PCE of 6.87%.^220^ A PANI‐based material, water‐soluble hydrochloric acid doped PANI (HAPAN) was also used as an ultrathin AIL with high transparency, small film roughness and suitable WFs.^221^ HAPAN‐based conventional OSCs based on poly(2‐ethylhexyl 6‐(4,8‐bis(5‐(2‐ethylhexyl)thiophen‐2‐yl)benzo[1,2‐b:4,5‐b′]­dithiophen‐2‐yl)‐3‐fluorothieno[3,4‐b]thiophene‐2‐carboxylate) (PBDTTT‐EFT):PC_71_BM achieved a high PCE of 9.05%. To search a strategy to create efficient hole‐collecting contacts on solution‐processed inverted OSCs, a series of thin p‐doped P3HT films were fabricated on undoped polymer layers.^222^

### 3.2 Metal Oxides and Metal Sulfides

Metal oxides and metal sulfides were used to substitute PEDOT:PSS as AILs in OSCs in recent years.^48^ Among these transition metal oxides, MoO_3_ is an important material as an AIL owing to its good transparency, high WF and much better environmental stability compared to PEDOT:PSS.

To reduce the cost, researchers have mainly focused on the solution‐possessed MoO*_x_* layers (s‐MoO_*x*_).^223^, ^224^, ^225^ By using s‐MoO*_x_* as the AIL, conventional OSCs based on P3HT:IC_70_BA showed better photovoltaic properties with a PCE of 6.57% and longer lifetime compared to the OSCs with a PEDOT:PSS AIL.^226^ It was revealed that the oxygen vacancy of the transition metal oxide played an important role in the electronic property, and the oxygen level could be controlled to improve the device performance.^224^ Through a sol–gel method, solution‐processed hydrogen molybdenum bronzes (HMBs) were prepared as AILs too.^227^, ^228^ HMBs with moderate reduction degree maintained high WFs and showed high densities of occupied gap states, both of which promote the hole‐transporting process. Compared to the solution‐processed MoO_3_ and PEDOT:PSS, the OSCs with the HMB AIL showed better performance with enhanced *J*_SC_, *V*_OC_ and *FF* values. As mentioned above, with their LSPR effects for enhanced light scattering, metal NPs are widely used to modify AILs including MoO_3_.^229^, ^230^ Using MoO_3_ covered Ag NPs to form an AIL, the conventional OSC exhibited 18% enhancement in PCE compared to the device with flat MoO_3_.^231^

OSCs based on VO*_x_* AILs were demonstrated to have good device performance.^232^, ^233^, ^234^ Solution‐processed VO*_x_* (s‐VO*_x_*) was prepared on a BHJ layer and was found to form conductive charge‐transport channels in the BHJ layer by a charge‐transfer doping reaction.^235^ Charge recombination was significantly reduced and high internal quantum efficiency of over 80% was achieved. Low‐temperature, solution‐processed V_2_O_5_ hydrate was explored with high stability,^236^ because water molecules can remain in the layered structure below 250 °C.

NiO*_x_* is another transition metal oxide which has been investigated as the AIL for OSCs.^237^ With a conduction band higher than the LUMO of organic donors and acceptors, NiO*_x_* is effective in blocking electrons and its high WF promotes the Ohmic contact at the interface between the BHJ and the anode. Based on a simultaneous treatment of thermal annealing and UV ozone, the processing temperature was significantly reduced to below 150 °C.^238^ The high concentration of NiOOH species on the film surface led to a large surface dipole which increased the WF. This NiO*_x_*‐based device showed good performance and stability. The relatively low temperature of NiO*_x_* also enables its fabrication on flexible substrates. Another method to reduce the processing temperature is to use colloidal NPs.^237^, ^239^ By room temperature preparation and without any post‐treatment, NiO*_x_* NPs AILs were used to fabricate high performance OSCs.^240^ With the PTB7‐Th:PC_71_BM BHJ blend as an active layer, the conventional devices showed an average PCE of 9.16%.

Other transition metal oxides such as WO*_x_* (H*_y_*WO_3–*x*_),^241^, ^242^ Fe_3_O_4_,^243^ CuO,^244^ RuO_2_,^245^ and CrO*_x_* 246 are also suitable for AILs in OSCs. Incorporating an ultrathin Ag film in Ormoclear/Ag/WO_3_ (OAW) structure to substitute ITO as transparent conducting electrode, the conventional OSCs exhibited an enhanced efficiency of 7.63% after surface modification.^247^ OAW exhibited low sheet resistance and high transmittance up to 96.3%. Solution processed s‐ReO*_x_* was also prepared as an AIL for efficient OSCs.^248^ Besides good hole‐transporting property, the s‐ReO*_x_* layer can change the light distribution in the active layer and enhance the absorption. The highest PCEs of s‐ReO*_x_*‐based conventional OSCs with P3HT:ICBA and PBDTTT‐C‐T:PC_71_BM were 7.26% and 8.30%, respectively.

Meanwhile, in some studies, metal sulfides were used as AILs too.^249^, ^250^, ^251^ Using UVO treatment to oxidize MoS_2_ into a MoS_2_/MoO_3_ double‐layered film as an AIL, efficient conventional OSCs based on P3HT:PCBM were fabricated with a PCE of 4.15%.^252^ The contents of the various valence states of molybdenum can be modified by the deposition temperature, which is correlated to the changed energy levels and optical/electrical properties. Ultrathin 2D MoS_2_ nanosheets prepared from chemical exfoliation showed excellent hole‐extraction property as an AIL in the PTB7:PC_71_BM‐based inverted OSC which exhibited a PCE of 8.11%.^253^

### 3.3 Graphene Oxides (GOs) and their Derivatives

Graphene oxide (GO) and its derivatives are efficient hole‐transporting materials in OSCs, with the advantage of solution processability, 2D structure, tunable electronic structures and low cost.^158^, ^254^ To improve the electron‐blocking ability, solution‐processed GO (sGO) was used to make the sGO/VO*_x_* and sGO/MoO*_x_* composite AILs.^255^ Meanwhile, the GO nanosheets can cover the active layer and prevent the penetration of the sol–gel precursors of the transition metal oxides into the active layer. As a result, the PCE and stability of OSCs with the composite AILs were both enhanced. The insulating nature of GO contributes to the increased series resistance of the corresponding OSC, thereby reducing the device performance. To improve the conductivity of GO, various approaches on post‐oxidation reduction have been made to remove the oxygen‐containing groups for recovering the conjugated structure of the basal plane.^256^, ^257^ Note that GO has a WF of ≈4.7 eV, which is much lower than the HOMO of organic donors and thus significantly influences the Ohmic contact of the active layer and the anode. Photochemical chlorination was found to be an efficient method to continuously tune the WF of GO to 5.21 eV.^258^ The chlorinated graphene oxide (Cl‐GO) films showed good optical transmission and hole‐transporting property as AILs for conventional OSCs. The resulting device based on poly[(4,8‐bis‐(2‐ethylhexyloxy)‐benzo[1,2‐b;4,5‐b′]dithiophene)‐2,6‐diyl‐*alt*‐(4‐(2‐ethylhexanoyl)‐thieno[3,4‐b]thiophene))‐2,6‐diyl] (PBDTTT‐C):PC_71_BM exhibited a PCE of 7.6%. Gold NPs were also utilized to modify the GO to take advantage of the LSPR effect.^259^, ^260^ Incorporating Au nanodisks (NDs) with nitrogen doped graphene (n‐G) as the AIL, conventional OSCs based on PTB7‐PC_71_BM:N‐MWCNTs, exhibited a 14.98% improvement in PCE (from 8.08% to 9.29%).^261^

### 3.4 Other Promising Materials

**Figure 12:**
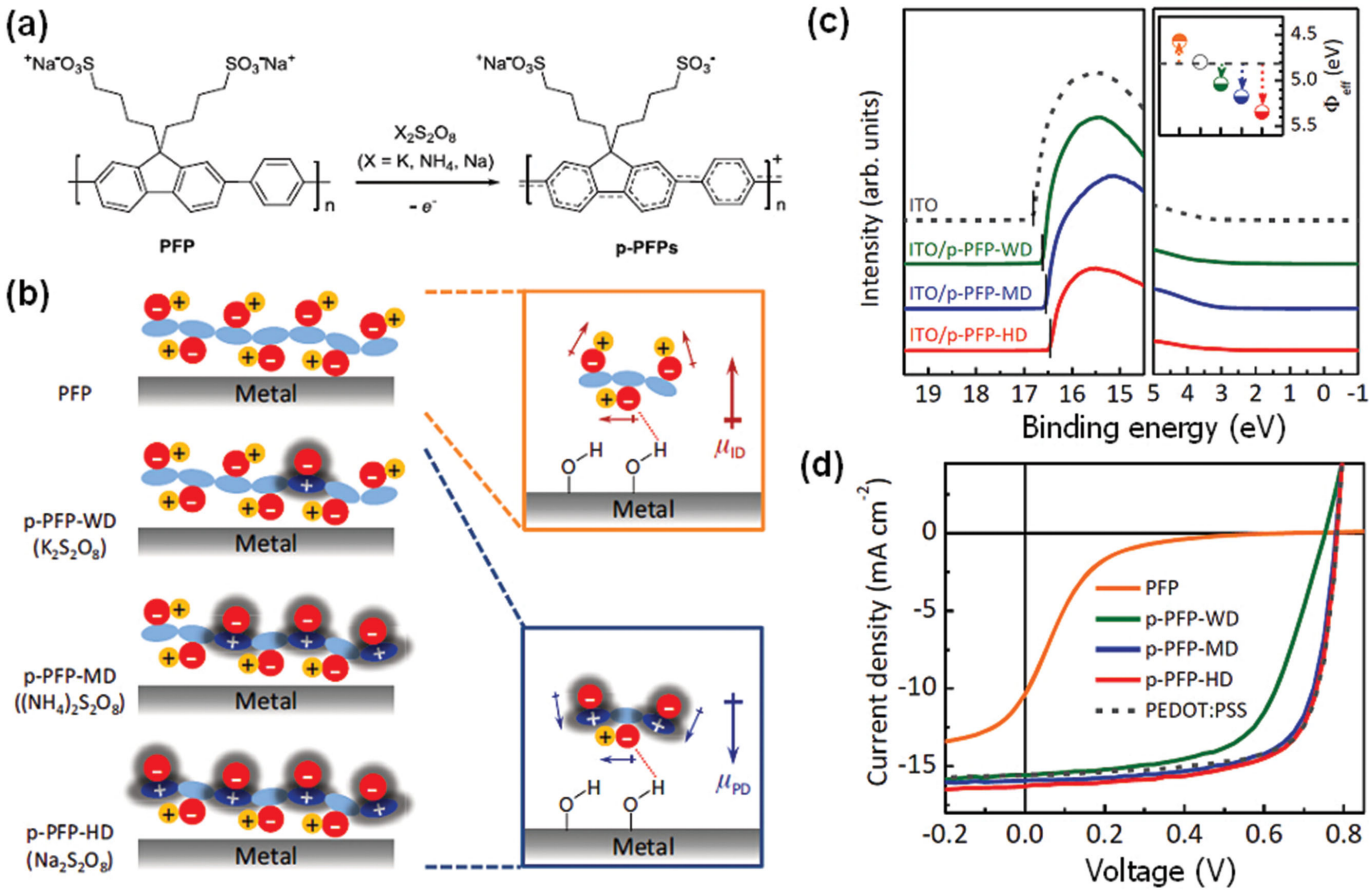
a) Schematic synthesis of p‐PFPs by oxidative treatments of *n*‐type PFP with persulfate salts. b) Illustration of p‐PFP configurations with varying degree of doping concentration (left) on metal electrodes and their plausible dipole formation (right) at interfaces between the electrode and *n*‐type PFP (right upper image) or p‐PFP (right lower image); sky blue: π‐conjugated backbone, red (−): alkyl side chain bearing a sulfonate functional group, blue (+): oxidized π‐conjugated backbone, yellow (+): a sodium counter ion, red dashed line: electrostatic interaction between sulfonate ion and hydrogen. The red and blue arrows represent the dipole direction of the ion‐induced dipoles (*μ*_ID_) and polaron‐induced dipoles (*μ*_PD_), respectively. c) Ultraviolet photoelectron spectroscopy spectra of the ITO with and without p‐PFPs. The inset displays the effective WFs of the ITO (open circle) coated with thin AILs of PFP (orange), p‐PFP‐WD (olive), p‐PFP‐MD (blue), and p‐PFP‐HD (red). d) *J–V* characteristics of the OSCs using different AILs. Reproduced with permission.^266^

Conjugated polyelectrolytes (CPEs) or small‐molecules as surface modifiers have been used to modify the WF of the anodes in OSCs.^262^, ^263^, ^264^, ^265^ Several *p*‐type CPEs (p‐PFP) with tunable WFs were developed by *p*‐doping of *n*‐type PFP [poly(9,9‐bis(4′‐sulfonatobutyl)fluorene‐*alt*‐*co*‐1,4‐phenylene)], as shown in **Figure** **12**.^266^ The doping process created new dipoles and the WFs of ITO shifted from 4.80 to 5.04–5.35 eV (Figure 12a–c). As a result, the conventional OSC using p‐PFPs as AILs exhibited an enhanced PCE up to 9.03% (Figure 12d) compared to the PEDOT:PSS‐based device (8.76%). The chlorobenzoic acid (CBA) has been used as an AIL with the advantages of solution processability and low‐cost.^267^ With the interfacial dipoles pointing toward the active layer induced by CBA, the WF of ITO was modified from 4.77 to 5.02 eV. Owing to the Ohmic contact by cascade energy level alignment, the conventional OSC device with CBA showed a highest PCE of 8.48%.

Other inorganic substances were also explored as hole‐transporting materials, such as CuI,^268^, ^269^ BiI_3_,^270^ and WC.^271^ Solution‐processed, UVO‐treated nickel acetate (O‐NiAc) has been demonstrated to be an efficient AIL.^272^ The dipole species NiOOH, which was partially decomposed from NiAc, led to the increased WF. After UVO treatment on the NiAc AIL, the conventional P3HT:ICBA‐based device exhibited an enhanced PCE of 6.64%. A metal complex of copper (I) thiocyanate (CuSCN) was also utilized as an efficient AIL in a recent report.^273^ Compared to the PEDOT:PSS‐based device, the CuSCN‐based device with a BHJ of poly(dithiophene‐diketopyrrolopyrrole‐2,5‐di‐2‐thienylthieno[3,2‐b]thiophene) (PDPP‐2T‐TT):PC_71_BM, exhibited a significantly enhanced PCE from 6.2% to 8.0%.

## 4 Interconnecting Layer (ICL) Materials for Tandem OSCs

With continuous efforts on material innovation and device optimization, great advances have been made in single‐junction OSCs over the past decade. In order to increase PCEs of single‐junction OSCs further, many low bandgap copolymers have been explored. However, most low bandgap copolymer‐based OSCs often output a high *J*_sc_ at the sacrifice of a respectable *V*_oc_, all of which prevent their further increases in PCE. To solve this problem, serial/parallel tandem devices were proposed to enhance the harvesting of a broader solar spectrum and diminish performance losses.^274^ In most of tandem OSCs, two or more subcells with complementary absorption are stacked and linked in series, offering a promising way to overcome the single‐junction limitations and improve PCEs. As a critical component in tandem OSCs to connect neighboring cells, interconnecting layer (ICL) materials are essential to the fabrication and operation of tandem devices. It is expected that good Ohmic contacts are formed between the ICL and subcells, where the ICL functions as a charge recombination zone to complete the circuit. Thus, the *V*_oc_ of tandem OSCs is estimated by summing the *V*_oc_s of individual subcells, while the overall *J*_sc_ is determined by the subcell with the smaller current. Ideally, ICLs should have good vertical conductivity to reduce electrical losses, high transmittance over the whole solar spectrum to minimize light absorption, appropriate energetic to facilitate charge recombination, full coverage to avoid subcell intermixing, and low surface roughness to avoid interrupting the deposition of adjacent subcells.^22^, ^275^ Therefore, sufficient material design and property control of ICLs is critical for tandem OSCs.^276^, ^277^, ^278^ The knowledge concerning interface sciences obtained from OPV and OLED studies provides a powerful support for ICL material design and property control in tandem devices. In this section, we present the developments on versatile ICL materials for high performance conventional and inverted tandem OSCs. The relationships between the ICL materials and device performance are comprehensively discussed.

### 4.1 ICL Materials for Conventional Tandem OSCs

In conventional tandem OSCs (CTOSCs), mostly designed ICLs can be categorized into two major configurations: electron transporting layer (ETL)–conducting layer–hole transporting layer (HTL) (e‐c‐h‐type) and ETL‐HTL (e‐h‐type). Both the ETL and HTL materials used in single‐junction OSCs can be used to develop ICL materials in tandem OSCs. Based on the different ETL, HTL and conducting materials, a variety of ICL materials were developed for CTOSCs. Device characteristics of some representative CTOSCs using various e‐c‐h‐type and e‐h‐type ICLs are summarized in **Table** **3**.

**Table 3:** Device characteristics of some representative conventional tandem OSCs using different ICLs

ICL configuration	Bottom cell	Top cell	*V*_OC_ (V)	*J*_SC_ (mA cm^−2^)	*FF* (%)	PCE (%)	Ref.
C_70_/BCP:C_60_/BCP/Ag/TAPC:MoO_3_/TAPC/DTDCTB	DTTz:C_70_	DTDCTB:C_60_	1.71	8.7	62	9.2	285
PTCBI/Ag/MoO_3_	DPSQ/C_70_	SubPc:C_70_	1.97	6.2	54	6.6	288
Bphen/Ag/MoO_3_	SubPc:C_70_	SubPc:C_70_	1.96	7.14	45	6.30	290
Bphen/Ag/F_16_CuPc/MoO_3_	SubPc:C_70_	SubPc:C_70_	2.00	7.38	52	7.66	290
C_60_/Bphen:C_60_/Ag/MoO_3_	DTDCTB:C_60_	DBP:C_70_	1.72 (2.58)a)	9.9 (7.3)a)	59 (59)a)	10.0 (11.1)a)	9
GO‐Cs/Al/GO/MoO_3_	PCDTBT:PCBM	PCDTBT:PCBM	1.69	5.03	46	3.91	291
LiF/ITO/MoO_3_	P3HT:bis‐PCBM	P3HT:PC_71_BM	1.14	6.14	73.7	5.16	292
TiO*_x_*/PEDOT:PSS	PCPDTBT:PCBM	P3HT:PC_71_BM	1.24	7.80	67	6.5	294
TiO_2_/m‐PEDOT:PSS	P3HT:ICBA	PSBTBT:PC_71_BM	1.47	7.6	63	7.0	296
Al/TiO_2_/PEDOT:PSS	P3HT:PC_71_BM	PSBTBT:PC_71_BM	1.25	7.44	63.2	5.84	295
ZnO/n‐PEDOT:PSS	GEN‐2:PCBM	pDPP5T‐2:PCBM	1.29	7.38	61	5.81	302
ZnO/PEDOT:PSS	PSEHTT:ICBA	PBDTTDPP:PC_71_BM	1.62	7.62	64.2	8.02	304
ZnO/n‐PEDOT:PSS	PCDTBT:PC_71_BM	PMDPP3T:PCBM	1.49 (2.09)a)	9.58 (7.34)a)	62 (63)a)	8.90 (9.64)a)	300
ZnO/n‐PEDOT:PSS	PTB7‐Th:PC_71_BM	PTB7‐Th:PC_71_BM	1.49	10.82	61	9.8	30
ZnO/PEDOT:PSS	SMPV1:PC_71_BM	PTTBDT‐FTT:PC_71_BM	1.56	8.45	64.3	8.48	305
LZO/PEDOT:PSS	SMPV1:PC_71_BM	PTTBDT‐FTT:PC_71_BM	1.38	6.83	61	5.75	305
ZnO/CPE‐K	PTB7‐Th:PC_71_BM	PTB7‐Th:PC_71_BM	1.51	11.05	64	10.6	30
ZnO/CPEPh‐Na	PTB7‐Th:PC_71_BM	PTB7‐Th:PC_71_BM	1.54	11.11	66	11.3	30
CPE1/CPE2/m‐PEDOT:PSS	SMPV1:PC_71_BM	SMPV1:PC_71_BM	1.82	7.70	72	10.1	306
ZnO/GO	SMPV1:PC_71_BM	PTTBDT‐FTT:PC_71_BM	1.46	8.33	50.0	6.07	305
ZnO/GO:SWCNTs	P3HT:PCBM	P3HT:PCBM	0.94	7.40	58	4.10	275
TiO*_x_*/GO	SMPV1:PC_71_BM	PTTBDT‐FTT:PC_71_BM	0.94	8.43	43.1	3.42	305
TiO*_x_*/GO/PEDOT:PSS	PSEHTT:ICBA	PSBTBT:PCBM	1.62	8.23	63.0	8.40	309

^a)^In the parentheses are device parameters of triple tandem OSCs.

#### 4.1.1 e‐c‐h‐type ICL Materials

For the e‐c‐h‐type configuration, the ICL materials are generally composed of a conductive layer sandwiched between an ETL and an HTL. The ETL and HTL are used for electron and hole collection, respectively, where the single intermediate conductive layer is served as charge recombination center. In fabricating CTOSCs, the optimized ICL electrically links two subcells, but it also extracts charge carriers from the front and back subcells and serves as a recombination zone for the collected carriers without a potential loss. Thus, the ICL is one of the most important components for the design of CTOSCs to maximize their performance.

At present, thin‐film metals (i.e., Au, Ag, Al) are usually adopted as highly conductive layer materials for most of e‐c‐h‐type ICLs. Using ETL/metal/HTL structures as the e‐c‐h‐type ICLs can fulfill all the requirements of high transparency, minimized voltage losses, and acting as an optical spacer to match the current between the subcells. Many thin metal layer based e‐c‐h‐type ICLs have been designed for small‐molecule or polymer CTOSCs, such as the C_60_/Au/zinc‐phthalocyanine,^279^ LiF/Al/Au/PEDOT:PSS,^280^ LiF/Al/WO_3_,^281^ LiF/Al/Au/MoO_3_,^282^ ZnO/Au/MoO_3_,^283^ and so on. Among them, the BCP/Ag/MoO*_x_* structure is one of commonly used ICLs in the small‐molecule CTOSCs due to its excellent optical transparency and the elimination of quasi‐Fermi level splitting at the interface between subcells.^284^, ^285^ Take a thermal‐deposited BCP/Ag/MoO_3_ ICL as an example, conventional multi‐tandem OSCs based on small‐molecule heterojunctions showed an extremely high *V*_OC_ of 5.89 V.^284^ This achieved high voltage can provide a power source for most consumer electronics which require a driving voltage of 5 V.

Different HTL materials used in the e‐c‐h‐type ICLs play an important role in determining device performance of CTOSCs. High WF HTL materials, such as ReO_3_, MoO_3_, and CuI with sequentially decreased charge generation efficiencies were employed to combine with BCP/Ag for designing various ICLs.^286^ Results showed that the charge generation efficiency of HTLs was an important parameter for high performance ICLs. The ICLs with higher charge generation efficiency of HTLs led to a higher *V*_OC_ due to the reduction of the difference between the Fermi level and HOMO level of the HTL, and a higher *FF* due to the efficient hole‐transport at the interface between Ag and HTL via the tunneling process. Note that ReO_3_ is the best HTL for this purpose among the designed ICL materials, where the use of ReO_3_ HTL in the ICLs resulted in negligible loss of *V*_oc_ and *FF* in the small‐molecule CTOSCs. Very recently, using a 1,1‐bis‐(4‐bis(4‐methyl‐phenyl)‐amino‐phenyl)cyclohexane (TAPC):MoO_3_ mixture as the HTL in e‐c‐h‐type ICLs offered flexibility in tuning the subcell position and thus maximizing *J*_SC_ of small‐molecule CTOSCs without sacrificing electrical properties.^285^ The TAPC:MoO_3_ HTL reduced contact resistances for hole extraction in the ICL to enhance the *FF*. The HTL also contributed to fine tuning of the spectral responses more effectively. As a result, an improved PCE of 9.2% for CTOSCs was achieved with a higher *FF* than that of the single‐junction subcells of 2‐((7‐(5‐(dip‐tolylamino)thiophen‐2‐yl)benzo[c]‐[1,2,5] thiadiazol‐4‐yl)methylene)malononitrile (DTDCTB):C_60,_ and 2‐((2‐(5‐(4‐(diphenylamino)phenyl)thieno[3,2‐b]thiophen‐2‐yl)thiazol‐5‐yl)methylene)malononitrile (DTTz):C_70_.

Different ETL materials used for the e‐c‐h‐type ICLs also act as a key role in determining device performance of CTOSCs. By using a C_60_:LiF nanocomposite ETL to replace the regular BCP material, a new e‐c‐h type ICL of C_60_:LiF/Ag/MoO*_x_* was used to achieve high *J*_SC_ and PCE in CTOSCs.^287^ The *J*_SC_ was enhanced by 40% after incorporating the nanocomposite ICL into the tandem device compared to the usual BCP/Ag/MoO*_x_* ICL, which was ascribed to the light absorption enhancement of the devices and the decreased energy barrier height at the interface between the ICL and the active layer. With an optimized ICL, consisting of 3,4,9,10‐perylenetetracarboxylic bisbenzimidazole (PTCBI) as an ETL, a nominal Ag layer, and a MoO_3_ HTL, a PCE of 6.6% was demonstrated for CTOSCs based on vapor‐deposited SubPc:C_70_ heterojunction and solution‐processed 2,4‐bis[4‐(N,N‐diphenylamino)‐2,6‐dihydroxyphenyl] squaraine (DPSQ)/C_70_ bilayer heterojunction.^288^ The high tandem *V*_OC_ of 1.97 V was equal to the sum of the subcells, indicating almost no loss for the transparent PTCBI/Ag/MoO_3_ ICL in charge collection/recombination between the subcells.

Replacement of the BCP with Bphen, a series of highly effective e‐c‐h‐type ICLs were designed to improve tandem cell performance by promoting the charge recombination, modulating the optical distribution and balancing photocurrents of each subcells within tandem devices.^289^ For instance, with a Bphen/Ag/MoO_3_ ICL, CTOSCs showing a PCE of 6.30% was obtained by stacking two SubPc:C_70_ BHJs.^290^ Using a modified ICL of Bphen/Ag/hexadecafluoro‐copper‐phthalocyanine (F_16_CuPc)/MoO_3_, a further improved PCE of 7.66% was achieved under the same subcells. Recently, Forrest et al. reported that the C_60_ (or C_70_)/Bphen:C_60_/Ag/MoO_3_ structures can be used as the efficient e‐c‐h‐type ICL to connect a subcell of DTDCTB blended with C_60_, and a tetraphenyl‐dibenzoperiflanthene (DBP):C_70_ subcell with ultraviolet‐to‐yellow absorbance.^9^ In the ICL, a 0.1 nm thick Ag NP layer was used for charge recombination and plasmonic field enhancement, while the C_60_/Bphen:C_60_ and MoO_3_ were utilized to collect electrons and holes, respectively. Correspondingly, the CTOSC delivered a PCE of 10.0%, which is >60% higher than the single cell efficiencies comprising the stack. To further achieve a higher *V*_OC_ and take the advantage of the optical field distribution, an additional DBP:C_70_ cell was inserted as the front subcell in the stack. The BPhen:C_60_‐based ICLs ensured minimal absorption loss between the subcells. The front and back green‐absorbing cells absorbed at different optical maxima to efficiently harvest short wavelength photons while complementing the absorption of the middle NIR‐absorbing cell. As a result, an improved PCE of 11.1% with an increased *V*_OC_ of 2.58 V was achieved for the triple‐junction device. This study suggests that multiple stacking e‐c‐h‐type ICLs by vacuum‐deposition are beneficial to fabricate high performance tandem/multijunction CTOSCs with complementary or non‐overlapping spectra.

Rapid developments on 2D carbon materials such as GO have also provided a new choice for developing e‐c‐h‐type ICLs in CTOSCs. Due to its salient properties and active functional groups, GO could be as a promising building block for ICL materials in tandem devices. For example, Dai et al. recently developed homo‐tandem PCDTBT:PCBM OSCs using GO‐based ICLs modified with ultrathin Al and MoO_3_.^291^ By using −COOCs groups to replace the periphery −COOH groups of GO via the charge neutralization, the WF of cesium‐neutralized GO (GO‐Cs) was reduced from 4.7 to 4.0 eV, matching well with the LUMO level of PCBM for efficient electron extraction/transportation. Meanwhile, the relatively weak light absorption of GO and GO‐Cs, together with their good solution‐processability for ultrathin film formation, facilitated the light transmission through the ICL to the rear cell. As a result, the tandem OSCs using the GO‐Cs/Al/GO/MoO_3_ ICL showed a significantly increased *V*_OC_ reaching ≈100% of the sum of the subcell *V*_OC_s, demonstrating a successful serial connection of subcells. The PCE obtained from the tandem OSC was 1.34 times that of the subcell.

Additionally, highly transparent and conductive metal oxides are good candidates as the single conductive layer in e‐c‐h‐type ICLs, owing to their high transmittance and facile processing. By designing multi‐layer structures of a highly conductive ITO layer sandwiched by a LiF ETL and a MoO_3_ HTL, a transparent conductive ITO‐based e‐c‐h‐type ICL was developed. In the ICL, the ITO layer was deposited by magnetron sputtering, and the LiF and MoO_3_ were deposited by vacuum evaporation.^292^ When used for the CTOSCs containing P3HT:bis‐PCBM and P3HT:PC_71_BM active layers, this ICL can improve the efficiencies of carrier transport in the active layers and the charge recombination within them. Resultantly, the tandem OSC exhibited an *FF* as high as 73.7%, leading to a greatly improved PCE of 5.16% compared to the single cells. However, future work should be focused on all solution process of this ICL and other similar ETL/metal oxide/HTL ICLs.

#### 4.1.2 e‐h‐type ICL Materials

Compared to the e‐c‐h‐type ICLs, the e‐h‐type ICLs have simpler material compositions, where a separate conductive layer is not needed between the ETL and HTL. Several e‐h‐type ICLs have been developed for polymer/small‐molecule OSCs, such as the Nb_2_O_5_/PEDOT:PSS,^54^ and Mg‐doped 4,7‐diphenyl‐1,10‐phenanthroline/MoO_3_.^293^ For these e‐h‐type ICLs, the HTL and ETL materials are joined together to function as a charge recombination zone. Thus, electrons collected by the ETL from the front cell combine with holes collected by the HTL from the back cell at the ETL‐HTL interface.

Currently, the mostly used e‐h‐type ICL materials for CTOSCs are based on the combination of an n‐type metal oxide (i.e, TiO*_x_*, ZnO, Nb_2_O_5_, etc.) and high WF PEDOT:PSS. In 2007, Kim et al. developed a transparent TiO*_x_*/PEDOT:PSS ICL for polymer CTOSCs with a landmark PCE of 6.5%.^294^ For such an ICL, highly conductive PEDOT:PSS (PH500) as an HTL material, was casted on a hydrophilic TiO*_x_* ETL material made by the sol–gel process, forming an e‐h‐type ICL of TiO*_x_*/PEDOT:PSS with negligible absorption. The ICL can effectively separate and link the front and back cells, leading to the completed tandem architecture. Because of its robustness, all‐solution processability, and function of good charge collection/recombination, this TiO*_x_*/PEDOT:PSS ICL opens a versatile design for the fabrication of efficient CTOSCs. Similarly, Yang et al. designed a robust ICL containing TiO_2_ nanocrystals combined with a commercial PEDOT:PSS to achieve efficient (5.84%) CTOSCs.^295^ Note that in these ICL materials, the PEDOT:PSS always have a relatively thin thickness, and metal oxides are normally not dense enough to protect the underlying layers (front cell) when solution‐processing the rear cell. The penetration of solvents through the ICLs may disturb their charge carrier selectivity, and result in charge recombination losses. Accordingly, reduced performance was widely observed from CTOSCs based on the ICL of TiO*_x_*/PEDOT:PSS. Designing modified‐TiO_2_ or modified‐PEDOT:PSS in ICLs is a good solution to tackle these problems. For instance, an effective ICL design of TiO_2_/m‐PEDOT:PSS was developed by incorporating sodium polystyrene sulfonate to modify PH500.^296^ When used for CTOSCs, this ICL broadened the solvent selection for the back active layer processing, so that each subcell can be prepared from its optimal solvent and deposition conditions. As a result, an improved PCE of 7.0% was demonstrated for the CTOSCs with the ICL. Utilizing acetylacetone coated TiO_2_ NPs, a new designed ICL consisting of highly dispersive TiO_2_ NPs as the ETL and conductive PH500 as the HTL was developed.^297^ The CTOSCs using this ICL delivered enhanced stability with respect to the single‐junction device due to the reduction of the oxygen diffusion into the bottom layer by the ICL.

Similar to the TiO*_x_*/PEDOT:PSS, ZnO/PEDOT:PSS is another important and efficient e‐h‐type ICL for CTOSCs. Many researches showed that a stack of solution‐processed ZnO NPs and pH‐neutral PEDOT:PSS (n‐PEDOT:PSS) was a general e‐h‐type ICL for various CTOSCs with good reproducibility.^298^, ^299^ Janssen et al. employed this ZnO/n‐PEDOT:PSS ICL to connect a wide bandgap BHJ of PCDTBT:PC_71_BM and a small bandgap BHJ of poly[[2,5‐bis(2‐hexyldecyl‐2,3,5,6‐tetrahydro‐3,6‐dioxopyrrolo[3,4‐c]pyrrole‐1,4‐diyl]‐*alt*‐[3′,3′′‐dimethyl‐2,2′:5′,2′′‐terthiophene]‐5,5′′‐diyl] (PMDPP3T):PCBM.^300^ The resulting conventional tandem and triple‐junction devices with high PCEs of 8.90% and 9.64%, respectively, were achieved due to their broad spectral responses up to 960 nm. Compared to the single‐junction counterpart, the PCEs of these CTOSCs were increased by as large as 50−60%. To obtain excellent functionality and reliability of this type of ICLs, Li and Brabec et al. developed a fully solution‐processed ICL comprising ZnO NPs and n‐PEDOT:PSS.^301^ With a low‐temperature (80 °C) drying in air, this ZnO/n‐PEDOT:PSS ICL (≈120 nm) had a good reproducibility and an overall transmittance of >90% in the range of 400–1000 nm, all of which made the ICL suitable for tandem, triple‐ and quadruple‐junction OSCs. Adopting a similar ZnO/n‐PEDOT:PSS ICL, this group also achieved fully printed CTOSCs which exhibited PCEs of 5.81% (on glass) and 4.85% (on flexible substrate) without *V*_OC_ losses,^302^ and efficient semitransparent CTOSCs which showed an average transmittance of 35.6%.^303^ Recently, Jang and co‐workers utilized a similar ICL to demonstrate all‐solution‐processed semitransparent CTOSCs with a high PCE of 8.02% and a large average transmittance of 44.9%.^304^ The high *V*_OC_ (1.62 V) for this CTOSC indicated the effectiveness of high‐performance ZnO/n‐PEDOT:PSS as the ICL. By contrast, using a solution‐processed TiO_2_:PEDOT:PSS ICL, the CTOSC with same subcells showed a much lower *V*_OC_ of 1.30 V and a decreased PCE of 4.76%. The device performance of the ZnO/n‐PEDOT:PSS ICL was also much better than that of the Ag/Al/Ca/MoO_3_ ICL (PCE = 5.11%) and the ZnO/Al ICL (PCE = 5.18%), which suffered from the problems such as chemical resistance and large interfacial energy losses. Very recently, this research group reported the use of ZnO/n‐PEDOT:PSS ICL for ITO‐free CTOSCs based on the subcells of (5E,5′E)‐5,5′‐((5′′,5′′′′‐(4,8‐bis(5‐(2‐ethylhexyl)thiophen‐2‐yl)benzo[1,2‐b:4,5‐b′]dithiophene‐2,6‐diyl)bis(3,3′′‐dioctyl‐[2,2′:5′,2′′‐terthiophene]‐5′′,5‐diyl))­bis(methanylylidene))bis(3‐octyl‐2‐thioxothiazolidin‐4‐one)­ (SMPV1):PC_71_BM and poly{4,8‐bis((2‐ethylhexyl)‐thieno[3,2‐b]­ thiophene)‐benzo[1,2‐b:4,5‐b′]dithiophene‐*alt*‐2‐ethyl‐hexyl‐4,6‐dibromo‐3‐fluorothieno[3,4‐b]thiophene‐2‐carboxylate}(PTTBDT‐FTT):PC_71_BM.^305^ The achieved PCE of this ITO‐free CTOSC with an electrode of Au‐doped single layer graphene nanoribbons was comparable to that of the commercial ITO‐based CTOSCs. The PCE dropped by 19.26% from its initial value (8.48%) after 840 h storage in air, showing good stability for the CTOSC without encapsulation. It is worth to note that the ICL plays a key role in determining the charge recombination process and the final device performance. Compared to ZnO/PEDOT:PSS ICL, the CTOSCs with ICLs of LZO/PEDOT:PSS, ZnO/GO, and TiO*_x_*/GO showed much lower PCEs of 5.75%, 6.07%, and 3.42%, respectively. This result was due to the large energy barrier, internal resistance, and inefficient charge extraction/recombination of the latter three ICLs, leading to a voltage drop in the ICLs and a decreased *FF* in the devices. This study proves that ZnO/PEDOT:PSS is an ICL that could prevent solvent penetration upon the deposition of the rear cell as it can be regarded as an excellent and robust ICL. A careful interface engineering and suitable selection of ICL materials is significant for achieving high performance CTOSCs.

In addition to the widely used PEDOT:PSS or metal oxide materials in the e‐h‐type ICLs, CPEs have recently been shown with some merits such as facile molecule design and synthesis, solution processability, chemical diversity, and mechanical robustness. The low WF CPEs can be designed as an alternative to *n*‐type metal oxide, while high WF CPEs can be used as an alternative to *p*‐type PEDOT:PSS. Thus, novel ICL materials comprising of CPE/PEDOT:PSS or *n*‐type metal oxide/CPE were developed to fabricate high performance tandem OSCs.

**Figure 13:**
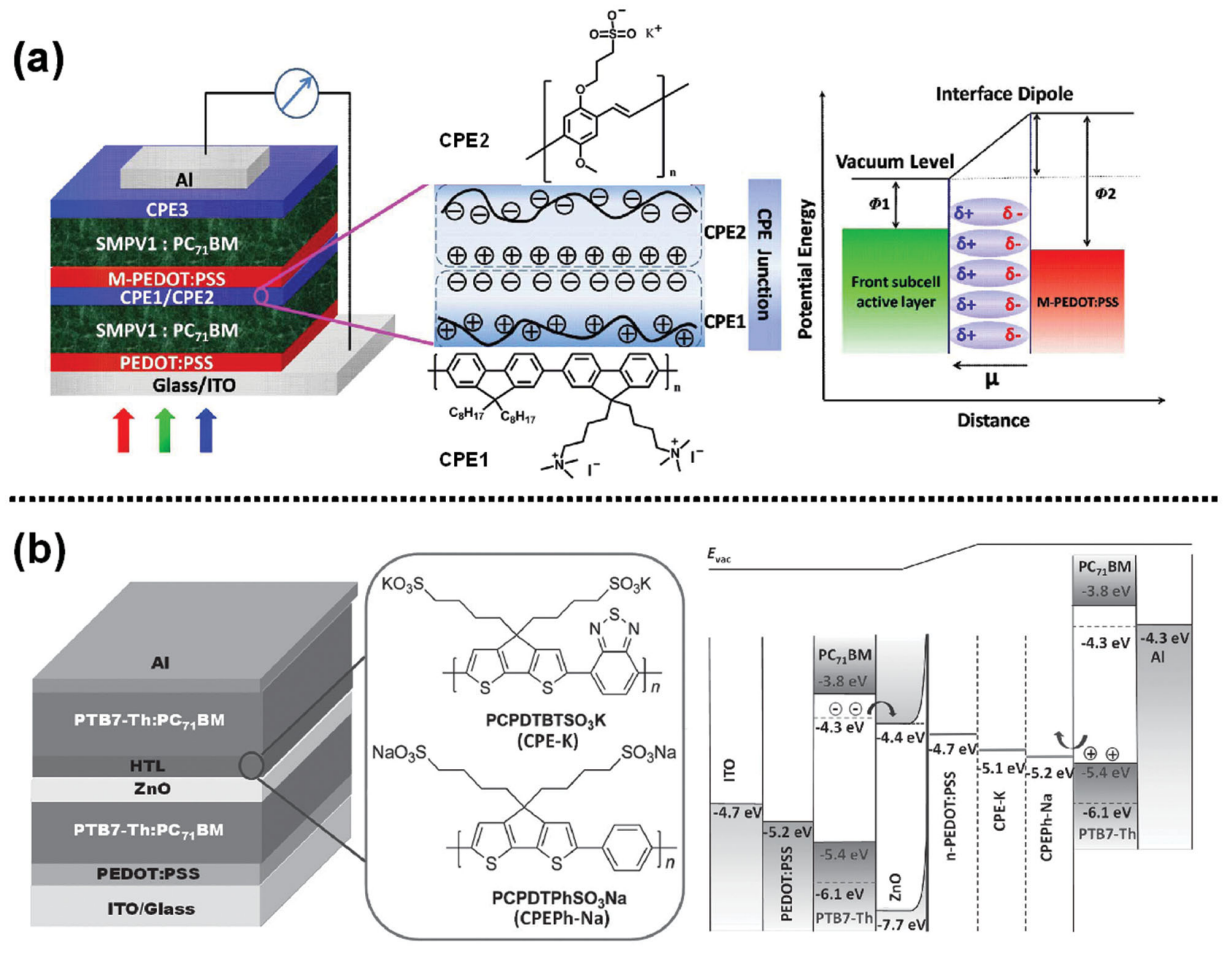
a) A schemetic tandem OSC with an e‐h‐type ICL of CPE1/CPE2/m‐PEDOT:PSS (left), the proposed scenario of self‐assembly bilayer CPE1/CPE2 (middle), and the schematic energy diagram (right) of the WF change due to the presence of CPEs between the front and back subcells. Reproduced with permission.^306^ Copyright 2013, Macmillan Publishers Limited. b) A schemetic tandem device with an ICL of ZnO/CPE (left), chemical structures of two HTLs (middle), and the energy diagram (right) of individual layers used in the tandem OSCs. Reproduced with permission.^30^

By using a solution‐processed bilayer structure of low WF CPEs combined with PEDOT:PSS as the e‐h‐type ICL, homo‐tandem OSCs with two subcells based on a 2D conjugated small molecule (SMPV1) were fabricated, exhibiting an improved PCE as high as 10.1% compared to 8.02% for the single‐junction device.^306^ In this new ICL, the polycation with a positively charged amine functional group (CPE1) was placed near the front cell, while the polyanion with a negatively charged –SO_3_^−^ functional group (CPE2) was used as the outermost layer (**Figure** **13**a). The self‐assembly of the bilayer CPE1/CPE2 created an internal polarization field that can shift up vacuum levels and reduce the electron‐injection barrier from the front cell to the middle ICL. The well‐organized multilayer charge film can realize a precise charge distribution and surface polarization, thus optimizing the charge collection from the front/rear cells and facilitating the charge recombination. The key features of this e‐h‐type ICL are thermal treatment‐free processing, which is crucial in maintaining the morphology of small‐molecule CTOSCs and offers a universal ICL candidate for other tandem devices. Very recently, highly self‐doped CPEs such as CPEPh‐Na and CPE‐K were demonstrated as effective *p*‐type HTLs to combine with ZnO ETL for solution‐processed tandem OSCs (Figure 13b).^30^ The WFs of HTLs play a significant role in the hole extraction and affect the charge recombination within the ICL. It was found that the CPEPh‐Na and CPE‐K HTLs showed high WFs of 5.20 and 5.15 eV, respectively, while both were much deeper than that of the n‐PEDOT:PSS (4.75 eV). Thus the ZnO/CPEs were found to be more efficient ICLs for the tandem devices because high WF CPEs work better than n‐PDEOT:PSS as HTLs. As a consequence, the ZnO/CPEs‐based OSCs showed better performance with enlarged *V*_OC_ and *FF*. The CTOSCs using ZnO/CPEPh‐Na and ZnO/CPE‐K ICLs exhibited high PCEs of 11.3% and 10.6%, respectively, better than that of the devices with the ZnO/n‐PEDOT:PSS ICL (PCE = 9.8%). The above results demonstrate a promising opportunity for universal applications of ICLs based on a metal oxide and highly self‐doped CPEs for all solution‐processed CTOSCs.

Additionally, several graphene‐ or GO‐based e‐h‐type ICL materials such as the graphene/MoO_3_,^307^ ZnO/GO:PEDOT:PSS,^308^ ZnO/GO:SWNTs,^275^ TiO*_x_*/GO/PEDOT:PSS,^309^ and the ZnO/GO and TiO*_x_*/GO^305^ were developed for CTOSCs. Although their device performance is mostly still worse than that of common e‐h‐type ICLs, these results suggest some alternative strategies for designing new ICLs based on 2D nanostructures as promising components in the future.

It is clearly seen that, significant advances in material design and property control of both e‐c‐h‐type and e‐h‐type ICLs have provided beneficial supports for achieving high performance CTOSCs. It is also worthwhile to note that, the latest developments on these ICL materials for constructing organic‐based hybrid tandem solar cells have emerged as a hot topic in energy related researches.^310^ Based on a newly designed PFN/TiO_2_/PH500/PEDOT:PSS ICL, a high PCE of 10.05% was reported for novel tandem solar cells consisting of a polymer:PCBM BHJ subcell and a perovskite subcell.^311^ Meanwhile, the use of AZO/Ag/MoO_3_, AZO/MoO_3_, or ITO/PEDOT:PSS as an ICL to connect the polymer:fullerene and *a*‐Si:H subcells, highly improved PCEs of 7.9 to 11.7% were achieved,^312^, ^313^ showing great potentials for future increase in efficiencies of organic/hybrid solar cells. We expect that the PCEs of CTOSCs as well as newly designed hybrid tandem systems could reach 15% or above when more efficient active materials and interfacial materials are developed and incorporated.

### 4.2 ICL Materials for Inverted Tandem OSCs

In the inverted tandem OSCs (ITOSCs), ICLs can also be classified into two main configurations: HTL‐conducting layer‐ETL (h‐c‐e‐type) and HTL‐ETL (h‐e‐type). Compared to the ICLs in CTOSCs, the ICL materials in the ITOSCs show a reversed stacking because the polarity of charge collection is the opposite of the conventional tandem device. Rapid developments on various HTL, ELT, and conducting materials have provided a great number of ICL materials for high performance ITOSCs. **Table** **4** summarizes the device characteristics of some representative ITOSCs based on different h‐c‐e‐type and h‐e‐type ICLs.

**Table 4:** Device characteristics of some representative inverted tandem OSCs with different ICLs

ICL configuration	Front cell	Back cell	*V*_OC_ (V)	*J*_SC_ (mA cm^−2^)	*FF* (%)	PCE (%)	Ref.
MoO_3_/Al/ZnO	P3HT:PCBM	PSBTBT:PC_71_BM	1.20	7.84	54.1	5.10	317
MoO_3_/Ag/ZnO/PFN	PDCBT:PC_71_BM	PBDT‐TS1:PCBM	1.60	11.65	54.5	10.16	11
MoO_3_/Ag/PFN	PIDT‐PhanQ:PC_71_BM	PTB7:PC_71_BM	1.60	9.95	68	10.98	319
PEDOT:PSS/Ag NW/ZnO	PCDTBT:PC_71_BM	pDPP5T‐2:PCBM	1.44	8.64	58	7.25	320
MoO_3_/m‐PEDOT:PSS/ZnO	PDTP‐DFBT:PC_71_BM	PDTP‐DFBT:PC_71_BM	1.36	11.5	65	10.2	321
WO_3_/PEDOT:PSS/ZnO	PTB7‐Th:PC_71_BM	PDTP‐DFBT:PC_71_BM	1.42 (2.28)a)	11.3 (7.63)a)	66.7 (63.4)a)	10.70 (11.55)a)	29
m‐PEDOT:PSS/ZnO	P3HT:ICBA	PDPP5T:PCBM	1.35	7.23	60	5.8	322
m‐PEDOT:PSS/ZnO	P3HT:ICBA	PDTP‐DFBT:PCBM	1.53	10.1	68.5	10.6	8
PEDOT/ZnO/Ba(OH)_2_	OPV12:PCBM	pDPP5T‐2:PC_71_BM	1.35	7.61	60	6.12	330
PEDOT/ZnO/PEI	GEN‐2:PCBM	PTB7‐Th:PC_71_BM	1.55	8.45	76.6	10.03	327
PEDOT:PSS/ZnO/FPQ‐Br	P2:PC_71_BM	PDPPTPT:PC_71_BM	1.60	8.4	64	8.58	326
m‐PEDOT:PSS/PH1000/ZnO/C_60_‐SAM	P3HT:ICBA	PCPDTFBT:PC_71_BM	1.57	7.83	66.5	8.2	328
n‐PEDOT:PSS/LZO/C_60_‐SAM	PSEHTT:ICBA	PTB7:PC_71_BM	1.54 (2.24)a)	10.30 (7.83)a)	65.5 (67.5)a)	10.39 (11.83)a)	31
Ag/PEDOT:PSS/ZnO NP	P3HT:ICBA	PTB7:PC_71_BM	1.49	7.38	71	7.81	331
P_2_Mo‐POM 18/ZnO	P3HT:ICBA	PTB7:PC_71_BM	1.59	9.05	69.0	9.9	37
PH1000/PEIE	P3HT:ICBA	PBDTTT‐C:PCBM	1.50	7.7	72	8.2	332
PEDOT:PSS/PEIE	PSEHTT:ICBA	PSBTBT:PC_71_BM	1.52	8.73	67.2	8.91	335
PEDOT:PSS/PEI	PTB7‐Th:PC_71_BM	PTB7‐Th:PC_71_BM	1.57	9.52	72	10.8	337
MoO_3_/Al‐doped MoO_3_	PCDTBT:PC_71_BM	PDPP3T:PC_71_BM	1.53	8.21	58.2	7.31	110
MoO*_x_*/Al_2_O_3_:ZnO/PEIE	P3HT:ICBA	PBDTTT‐C:PCBM	1.48	7.1	62	6.5	338

^a)^In the parentheses are device parameters of triple tandem OSCs.

#### 4.2.1 h‐c‐e‐type ICL Materials

In general, most of h‐c‐e‐type ICLs are based on HTL/metal/ETL materials. When used for ITOSCs, thin‐film of Ag or Al as the single conductive layer in the ICL functions as a recombination center for hole and electron carriers, which can be extracted/collected from the front and back subcells by the HTL and ETL, respectively. Therefore, each component material used in the h‐c‐e‐type ICLs will directly influence charge collection and recombination processes, finally determining the overall device performance.

Earlier reports on HTL/metal/ETL materials demonstrated that a thermally evaporated MoO_3_/Ag/Al/Ca multilayer is an effective h‐c‐e‐type ICL to connect two inverted subcells of P3HT:PCBM and to realize an Ohmic contact between the front and back cells for good charge extraction/recombination.^314^ However, the use of a reactive metal such as Ca is not desirable in the inverted device. To solve it, by replacing Ca with metal oxide to promote electron‐collection and hole‐blocking, some alternative h‐c‐e‐type ICLs of HTL/metal/metal‐oxide ETL structures such as MoO_3_/Ag/Al/ZnO,^315^ MoO_3_/Au/ZnO,^283^ and PEDOT:PSS/Au/TiO*_x_* 316 were developed to improve the performance of ITOSCs. Based on a P3HT:PCBM front cell and a poly[(4,4′‐bis(2‐ethylhexyl)dithieno[3,2‐b:2′,3′‐d]silole)‐2,6‐diyl‐*alt*‐(2,1,3‐benzothiadiazole)‐4,7‐diyl] (PSBTBT):PC_71_BM back cell, efficient ITOSCs with a PCE of 5.1% were fabricated by using the optimized MoO_3_/Al/ZnO ICL.^317^

Meanwhile, low WF polyelectrolytes such as PEIE and PFN can also be used as solution‐processed ETL materials to reduce the WF of the MoO*_x_*/metal or MoO_3_/Ag/ZnO in the h‐c‐e‐type ICLs, by which high performance ICLs of MoO*_x_*/Ag/PEIE, MoO_3_/Ag/PFN, and MoO_3_/Ag/ZnO/PFN were developed for efficient ITOSCs.^11^, ^318^, ^319^ With the MoO_3_/Ag/ZnO/PFN ICL, high efficiency ITOSCs were recently fabricated by Hou et al. using a PDCBT:PC_71_BM bottom cell and a PBDT‐TS1:PCBM top cell.^11^ In this h‐c‐e‐type ICL, MoO_3_ and PFN‐modified ZnO were respectively selected as the HTL and ETL, while an Ag (0.5 nm) layer was added to establish an Ohmic contact between MoO_3_ and ZnO, and to change the wettability of MoO_3_ surface which stimulated localized plasmonic enhancement effects. A resulting PCE as high as 10.16% (a certified value of 9.59%) for the ITOSC was achieved with good reproducibility. This ITOSC can maintain almost 80% of the initial PCE after 100 days, suggesting its good ambient stability. Moreover, Chen and Jen et al. designed a versatile ICL based on a thin Ag (8–14 nm) film sandwiched between a MoO_3_ HTL and a PFN ETL, which enable the resulting ITOSCs to produce a high PCE up to 10.98% and an external quantum efficiency greater than 90%.^319^ The excellent performance was attributed to the fact that the optical field distribution can be optimized via strong reflectivity formed by the Ag layer to obtain balanced currents between the two subcells. For another, the thin Ag reflecting mirror also allows light to be confined well in the tandem device due to the generation of a micro‐cavity in the back cell.

**Figure 14:**
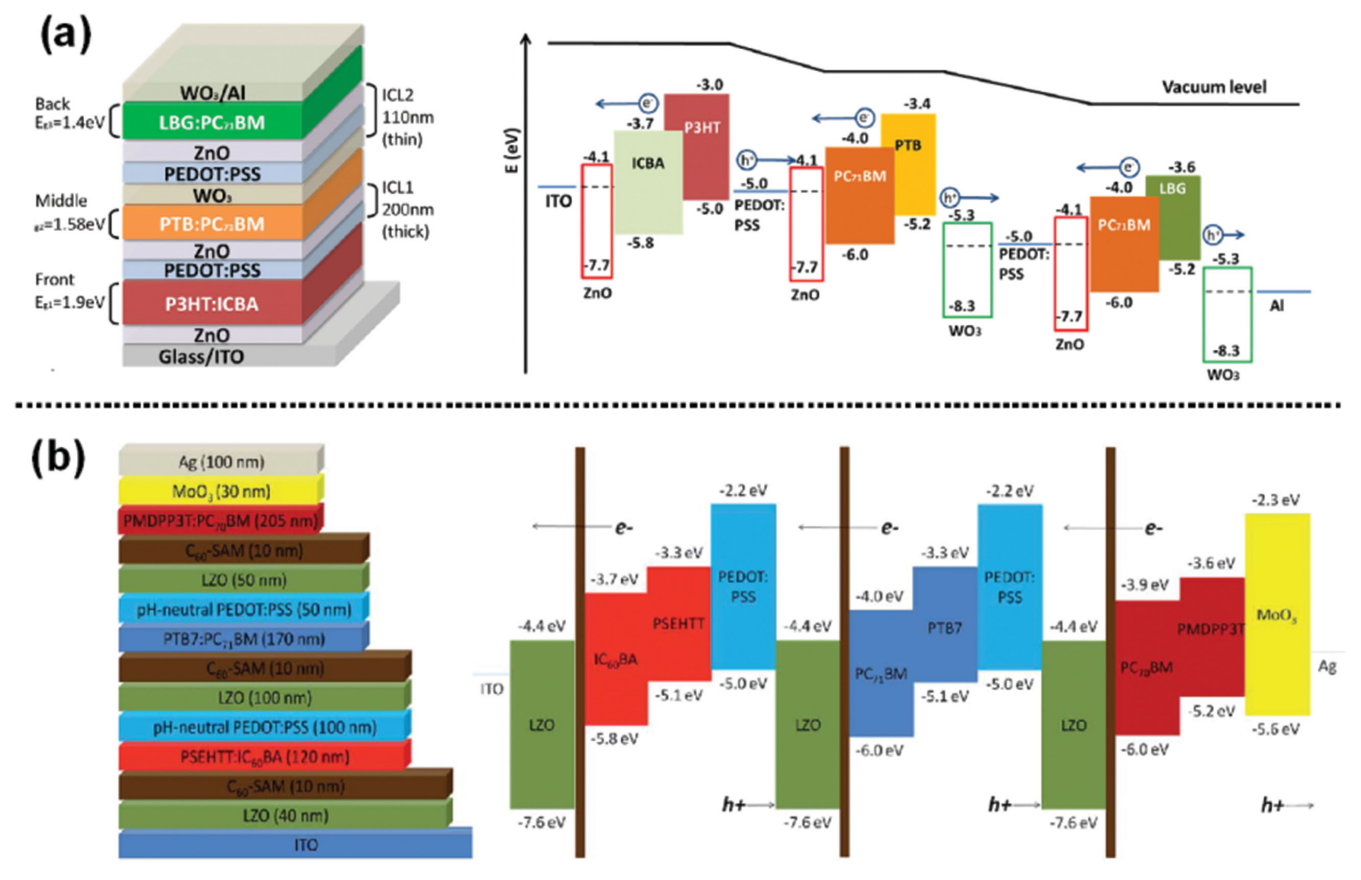
a) The triple tandem OSC using a h‐c‐e‐type ICL of WO_3_/PEDOT:PSS/ZnO (left) and the relevant energy level diagram (right). LBG represents PDTP‐DFBT, and PTB represents PTB7‐Th. Reproduced with permission.^29^ b) The triple tandem device with a h‐e‐type ICL of n‐PEDOT:PSS/ZnO/C_60_‐SAM (left) and the corresponding energy level diagram (right). Reproduced with permission.^31^ Copyright 2015, Royal Society of Chemistry.

Although high efficiency ITOSCs have been achieved by utilizing several h‐c‐e‐type ICLs based on the evaporated metal layer, developing all‐solution processed h‐c‐e‐type ICL materials is still a challenge. Thus, a solution‐processed metal or metal‐like conductive layer is proposed to achieve all‐solution processable h‐c‐e‐type ICLs for high performance ITOSCs. For example, solution‐processed Ag NWs were packed between HTL and ETL materials to realize fully functional, solution‐processed h‐c‐e‐type ICLs, such as PEDOT:PSS/Ag NW/ZnO and WO_3_/Ag NW/ZnO.^320^ These Ag NW‐based ICL materials were found to be of Ohmic nature under an applied bias, which provided efficient and robust ICLs for improving charge recombination properties in ITOSCs. With an optimized PEDOT:PSS/Ag NW/ZnO ICL, a high PCE of 7.25% was achieved for the resulting ITOSCs based on the PCDTBT:PC_71_BM bottom cell and the diketopyrrolopyrrole–quinquethiophene alternating copolymer (pDPP5T‐2):PCBM top cell. Besides Ag NWs, solution‐processed PEDOT:PSS were adopted as a metal‐like layer between the metal oxide HTL and ETL as a recombination center and also as a protecting layer to develop h‐c‐e‐type ICLs by all‐solution processing.^29^, ^321^ The high conductivity of PEDOT:PSS can reduce contact resistance between the metal oxide HTL and ETL because the free charge concentration in the metal oxides is not high enough. Meanwhile, the robust PEDOT:PSS can effectively avoid solvent penetration into the bottom layer during coating top layer because metal oxide films usually act as thin interface layers with very small thickness for high charge selectivity. Using a designed MoO_3_/m‐PEDOT:PSS/ZnO ICL, the ITOSCs with identical subcells showed an enhanced PCE of 10.2% (vs 8.1% for the single‐junction device) for the poly[2,7‐(5,5‐bis‐(3,7‐dimethyloctyl)‐5H‐dithieno[3,2‐b:2′,3′‐d]pyran)‐*alt*‐4,7‐(5,6‐difluoro‐2,1,3‐benzothiadiazole)] (PDTP‐DFBT):PC_71_BM system.^321^ Likewise, by inserting a heavily doped PEDOT:PSS between WO_3_ HTL and ZnO ETL, a newly designed h‐c‐e‐type ICL of WO_3_/PEDOT:PSS/ZnO was used to achieve efficient double and triple‐junction ITOSCs.^29^ In this all‐solution‐processed ICL, WO_3_ NPs with a conduction band of 5.3 eV acted as the HTL to extract holes from the PTB7‐Th:PC_71_BM subcell, while the ZnO with a conduction band of 4.1 eV served as the ETL to extract electrons from the PDTP‐DFBT:PC_71_BM subcell. Between WO_3_ and ZnO, the PEDOT:PSS functioned as a charge recombination layer for the opposite charge carriers from adjacent subcells to tunnel through and cancel out. Thus, the quasi‐Fermi energy level of donor in a subcell can be aligned with the quasi‐Fermi energy level of acceptor in the other, leading to a downward shift of the vacuum level, as reflected in the energy level diagram (**Figure** **14**a). As a result, the double and triple junction devices using this ICL delivered excellent PCEs up to 10.70% and 11.55%, respectively.

#### 4.2.2 h‐e‐type ICL Materials

As mentioned above, most of h‐c‐e‐type ICLs suffer from complicated evaporation steps and possible optical losses induced by the metal layer. Many efforts have thus been focused on h‐e‐type ICLs which have fewer components with respect to the h‐c‐e‐type ICLs. In the h‐e‐type ICLs, they are usually comprised of a first HTL material for hole‐collection, and a second ETL material for electron‐collection, where the charge recombination is completed at the ETL‐HTL interface. The simple material compositions and easy solution‐processing of the h‐e‐type ICLs make them very promising ICL materials for ITOSCs with high PCEs and good stability.

Similar to the e‐h‐type ICLs, one of the most popular designs for the h‐e‐type ICLs is based on a combination of all‐solution‐processed PEDOT:PSS and ZnO. In the PEDOT:PSS/ZnO ICL, the high WF PEDOT:PSS serves as a HTL to extract/collect holes from the front cell, while ZnO acts as an ETL to assure an efficient electron‐collection from the back cell. Several works have shown the generation of an equivalent Ohmic interface between the highly conductive m‐PEDOT:PSS and ZnO NPs, which promises an efficient and robust ICL for various ITOSCs.^322^, ^323^ By using the m‐PEDOT:PSS/ZnO ICL to connect two subcells with complementary absorption, high efficiency ITOSCs were successfully achieved in a series of wide‐ and low‐bandgap polymer‐based BHJs.^8^, ^324^, ^325^ A good electrical/optical coupling of two subcells based on P3HT:ICBA and PDPP5T:PCBM was realized by using a fully solution‐processed m‐PEDOT:PSS/ZnO ICL, leading to a PCE of 5.8% for the ITOSCs.^322^ With a similar m‐PEDOT:PSS/ZnO ICL, Yang et al. reported ITOSCs with a landmark PCE of 10.6%, where the P3HT:ICBA bottom cell and PDTP‐DFBT:PCBM top cell have complementary absorption bands.^8^ The achieved PCE is the first certified OSC efficiency over 10%, which indicates a great potential of the all‐solution‐processed PEDOT:PSS/ZnO ICL for fabricating high performance ITOSCs.

Based on the PEDOT:PSS/ZnO materials, some new designed ICL materials are further developed to control over their energy level alignments and interfacial properties, in order to improve the device performance of various ITOSCs. For example, using a CPE, poly(9,9′‐bis(6′′‐N,N,N‐trimethylammoniumhexyl)fluorene‐*co‐alt*‐phenylene) with bromide counter ions (FPQ‐Br) to modify ZnO NPs as the ETL, a novel h‐e‐type ICL of PEDOT:PSS/ZnO/FPQ‐Br was successfully used to improve the performance of ITOSCs based on a front subcell of thieno[3,4‐c]pyrrole‐4,6‐dione‐terthiophene copolymer (P2):PC_71_BM and a back subcell of PDPPTBT:PC_71_BM.^326^ The FPQ‐Br modification not only smoothed the ZnO surface, but also reduced its WF from ≈4.7 to 4.3 eV. The decreased WF can lower the energy barrier between PDPPTBT:PC_71_BM layer and ZnO, which enables it more efficiently to collect electrons, consequently enhancing the device efficiency from 7.23% to 8.04% and further to 8.58% (optimized). Very recently, by inserting a thin PEI layer on the ZnO‐coated PEDOT to reduce charge recombination at the surface of the back cell, air‐processed ITOSCs incorporating GEN‐2:PCBM as a front cell and PTB7‐Th:PC_71_BM as a back cell delivered a PCE up to 10.03% with a high *FF* of 76.6%.^327^ A fullerene self‐assembled monolayer (C_60_‐SAM) can also be used to passivate ZnO surface traps and enhance electric coupling between ZnO and the back organic active layer, therefore forming an effective h‐e‐type ICL of PEDOT:PSS/ZnO/C_60_‐SAM for opaque/semitransparent ITOSCs.^328^ The PEDOT:PSS/high conductivity PEDOT:PSS (PH1000))/ZnO/C_60_‐SAM ICL was also successfully used to connect various subcells due to its combined properties including high transmittance (≈85% in 700–900 nm), reasonable conductivity (≈15 S cm^−1^), smooth surface, and good robustness against solvent erosion.^329^ As a result, high PCEs up to 8.5% and 7.8% were obtained for opaque and semi‐transparent ITOSCs, respectively. The high tandem *V*_OC_ values were equal to the summed *V*_OC_ of each subcells, further confirming the effectiveness of this type of ICLs.

**Figure 15:**
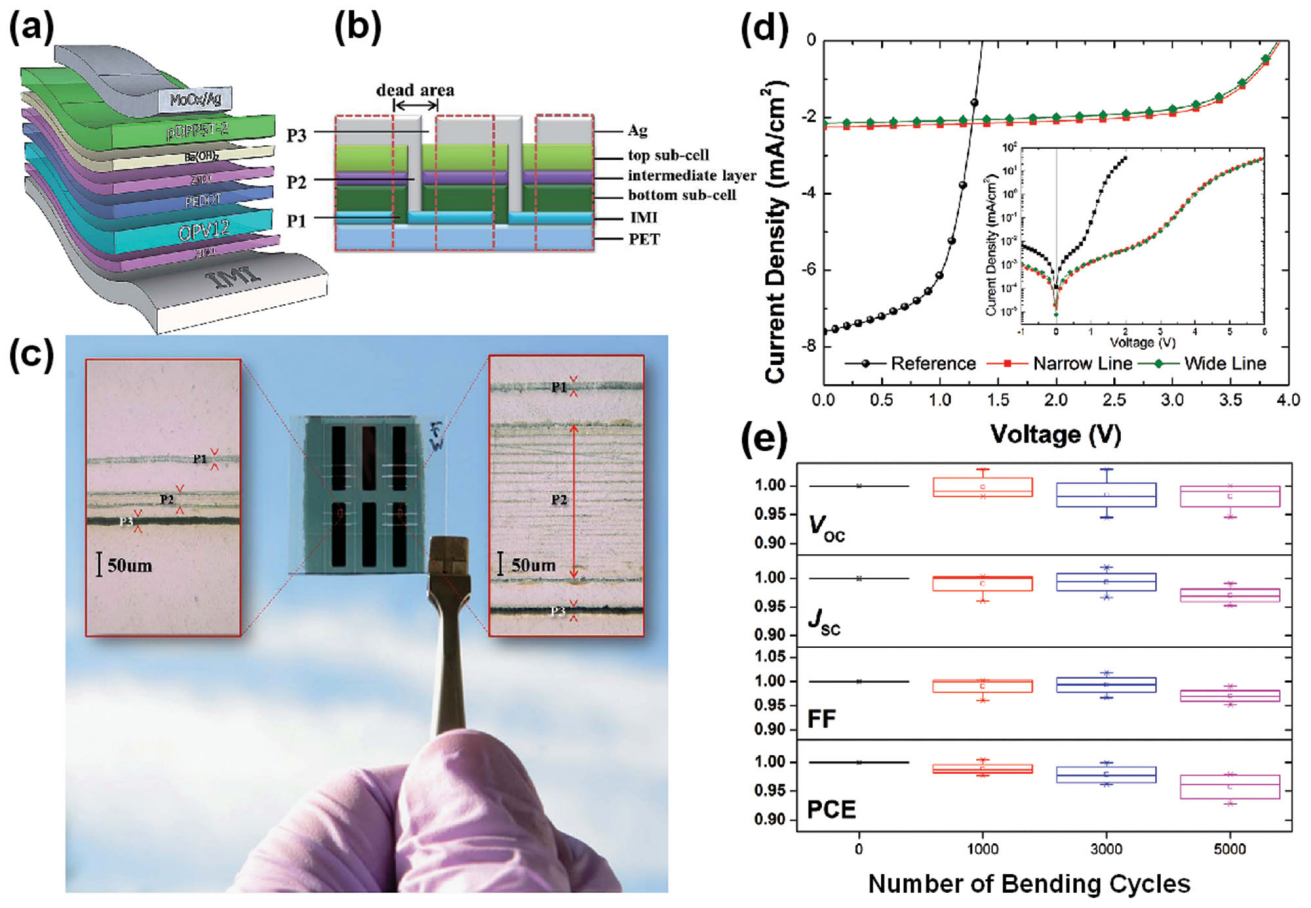
a) A schematic structure of flexible ITOSCs using the PEDOT/ZnO/Ba(OH)_2_ ICL. b) Schematic illustration of the interconnection lines in the organic tandem module (3 cells module). c) Photograph of one of the 9 substrates carrying two reference single tandem cells (center) and two pairs of tandem modules (left and right), with narrow (≈25 μm, left) and wide (≈325 μm, right) P2 line patterning. The insets present top views from an optical microscope showing the lines P1–P3. The wide P2 line was realized by laser hatching (scanning many single lines parallel to each other). d) *J–V* characteristics of the reference ITOSC and tandem modules with narrow (≈25 μm) and wide (≈325 μm) P2 lines under illumination. The inset shows the dark *J–V* characteristics. e) Normalized device characteristics of flexible tandem modules after 1000, 3000 and 5000 bending cycles. Reproduced with permission.^330^ Copyright 2014, Royal Society of Chemistry.

By modifying ZnO surface with a thin electron‐selecting Ba(OH)_2_ layer, Li and Brabec et al. designed an all‐solution‐processed PEDOT/ZnO/Ba(OH)_2_ ICL.^15^ This Ba(OH)_2_ modified h‐e‐type ICL (≈60–80 nm) showed an excellent transmittance in the range of 400–950 nm, allowing it to act as an efficient ICL for ITOSCs based on both the glass (PCE = 7.66%) and the flexible (PCE = 5.56%) substrates. Using this PEDOT/ZnO/Ba(OH)_2_ ICL (a thickness of ≈80 nm), Spyropoulos and co‐workers successfully developed high‐performance flexible ITOSCs and tandem modules.^330^ As depicted in **Figure** **15**, the ITO‐metal‐ITO (IMI)–coated PET‐based ITOSC is made up of a low bandgap pDPP5T‐2:PC_71_BM top cell and a moderate bandgap OPV12:PCBM bottom cell. Owing to the good connection and charge collection/recombination effects of this efficient and robust ICL, the flexible ITOSC delivered a high PCE of 6.12% with a *V*_OC_ of 1.35 V. Furthermore, flexible tandem modules by interconnecting three single ITOSCs in series were fabricated. The architecture of the whole module is schematically illustrated in Figure 15b, where the interconnection procedure consists of laser ablation of three patterning lines P1–P3. Figure 15c shows a photograph of one of the substrates and optical microscopy images capturing the P1–P3 lines. *J–V* characteristics of a representative reference ITOSC and two corresponding tandem modules are shown in Figure 15d. The flexible tandem modules showed very low interconnection resistance in comparison with the single ITOSC and exhibited an outstanding PCE up to 5.7% with a *V*_OC_ output of 3.9 V. Meanwhile, compared to the tandem module with a wide (≈325 μm) P2 line, the tandem module with a narrow (≈25 μm) P2 line performed slightly better due to the smaller dead area, suggesting the benefit of a laser‐controlled patterning way for dividing tandem cells into tandem modules. In both cases, the P2 line was fully functional and the devices showed similarly high interconnection quality, as confirmed by the *J–V* performance (Figure 15d). Figure 15e presents normalized changes in photovoltaic performance throughout 5000 bending cycles for the tandem modules. A small loss in PCE in the range of only 2–7% was observed, demonstrating high mechanical resilience for this type of devices which is benefited from the thick/robust ICL materials, the flexible active layers and electrodes. The high mechanical resilience of the high efficiency tandem modules suggests that this type of flexible ITOSCs could potentially be applied as a power source on non‐planar, foldable and moving surfaces.

Besides the surface modification, bulk doping of ZnO by metal elements (i.e., In, Al and Li) is another good route to improve electron‐conductivity of ZnO‐based ETLs, therefore providing beneficial supports in designing high performance ICLs of PEDOT:PSS/ternary oxide (such as IZO, AZO, and LZO) for ITOSCs with improved PCEs and stability.^31^, ^45^, ^105^ When a combination of AZO and PEODT:PSS (Al 4083) with a total thickness of ≈160 nm was developed for ITOSCs, the ICL showed good electrical and optical properties. Thus the film can serve as efficient and robust ICL as well as an optical spacer for tandem and multi‐junction devices.^45^ By the use of a h‐e‐type ICL based on the n‐PEDOT:PSS HTL and the LZO/C_60_‐SAM ETL, high PCEs of 10.30% and 11.83% was achieved for the ITOSCs with tandem and triple‐junctions (Figure 14b), respectively.^31^ The 11.83% efficiency is a record high value for OSCs reported to date, further demonstrating that the inverted tandem structure based on the n‐PEDOT:PSS/LZO/C_60_‐SAM ICL is a powerful and promising way to realize high performance OSCs.

Additionally, to improve the wettability of aqueous PEDOT:PSS HTL on a hydrophobic active layer, an ultra‐thin Ag layer (subnano‐scale) was inserted between the bottom P3HT:ICBA active layer and the PEDOT:PSS/ZnO ICL in the ITOSC with a top subcell of PTB7:PC_71_BM.^331^ The PCE was improved from 7.06% without Ag to 7.81% with 0.5 nm Ag layer for a small‐area (0.03 cm^2^) ITOSC, and from 2.19% to 6.11% for a large area (1 cm^2^) ITOSC. The enhanced performance and the allowed larger area device can be ascribed to more efficient hole collection and reduced leakage current for the bottom cell with improved film forming of PEDOT:PSS. This result suggests that high‐quality PEDOT:PSS film is of importance for achieving large‐area ITOSCs with good performance.

In addition to the widely PEDOT:PSS/ZnO‐based ICLs, several h‐e‐type ICLs have also been developed for efficient ITOSCs by replacing the PEDOT:PSS HTL, or the ZnO ETL, or both of them with new HTL or ETL materials. Note that the use of PEDOT:PSS (4.9 eV) in combination with ZnO (a typical WF around 4.3 eV), limits the WF contrast between the two surfaces of the h‐e‐type ICL to less than about 0.6 eV, consequently confining the range of active layer materials that can be used to assemble ITOSCs. To overcome the limitations, several solution‐processable ETL materials with lower WFs, such as NCPEs, and copper‐phthalocyanine derivatives were used as alternatives to ZnO for fabricating high performance ITOSCs.^332^, ^333^, ^334^ For instance, Kippelen et al. developed a new ICL comprising NCPE HTL (PEIE or PEI) and PH1000 (high conductivity PEDOT:PSS) layers, which have been shown to display a large WF contrast (1.2 eV) between its two interfaces with the bottom and top active layers in the ITOSCs.^332^ Using PEIE or PEI, the designed ICLs exhibited a WF of ≈3.6 eV, which is low enough to produce efficient electron‐collecting interfaces. With the PH1000/PEIE ICL, tandem devices based on the subcells of P3HT:ICBA and PBDTTT‐C:PCBM, displayed a high PCE of 8.2% with an *FF* of 72%. Using a similar PEDOT:PSS/PEIE ICL for connecting broaden light‐absorbing subcells, a further improved PCE up to 8.91% with good stability was demonstrated for ITOSCs with the bottom cell (PSEHTT:ICBA) and the top cell (PSBTBT:PC_71_BM).^335^ In contrast, the ITOSCs using other h‐e‐type ICLs of PEDOT:PSS/ZnO, n‐PEDOT:PSS/ZnO, and graphene/TiO*_x_* showed smaller *V*_OC_s of 1.4, 1.2, and 1.12 V, and thus much lower PCEs of 8.30%, 6.04%, and 5.11%, respectively. Combining PEDOT:PSS with NCPEs as a recombination center, the presence of dual WFs on two sides and the formation of tunnel junctions in the PEDOT:PSS/NCPE ICL resulted in high‐performance ITOSCs with a PCE of 8.32%.^336^ Very recently, Lee et al. reported a one‐step nanocomposite solution process to design a self‐organized PEDOT:PSS/PEI ICL, which was used to construct a simplified four‐layer tandem devices.^337^ A high PCE up to 10.8% for the ITOSCs was resulted from the excellent features of this self‐organized h‐e‐type ICL including high optical transparency, low electrical resistance, large WF offset, and favorable Ohmic contacts at the interfaces with the subcells (PTB7‐Th:PC_71_BM). These studies based on polymeric h‐e‐type ICLs are very effective and universal to improve performance of various ITOSCs and also show great potentials towards large area fabrication and high productivity such as roll‐to‐roll processing of inverted tandem OSCs.

On the other hand, high WF MoO*_x_* is also a good alternative to PEDOT:PSS in designing effective h‐e‐type ICLs for ITOSCs. For example, physically robust ICLs based on a MoO_3_ HTL and an Al‐doped MoO_3_ ETL made by co‐evaporation were developed to achieve efficient ITOSCs.^110^ In this type of h‐e‐type ICLs, Al‐doping of top half MoO_3_ layer reduced the high WF MoO_3_ into low WF Al‐doped MoO_3_, and thus provided a large WF offset at both sides of the MoO_3_‐based ICL without sacrificing its high transparency. Correspondingly, ITOSCs using two complementary absorbing BHJ cells of PCDTBT:PC_71_BM and PDPP3T:PC_71_BM showed a PCE of 7.31%, suggesting high charge collection and recombination efficiencies in a tandem structure with this ICL. Based on a HTL of thermally deposited MoO*_x_*, MoO*_x_*/Al_2_O_3_:ZnO nanolaminate/PEIE, MoO*_x_*/Al_2_O_3_:ZnO nanolaminate, or MoO*_x_*/ZnO/PEIE was used as a h‐e‐type ICL for ITOSCs based on two active layers of P3HT:ICBA and PBDTTT‐C:PCBM with complementary absorption ranges^338^ Using the highly conductive Al_2_O_3_:ZnO nanolaminate with PEIE modification delivered about 20% higher *FF* than the ICL containing ZnO modified by PEIE, finally leading to an improved PCE of 6.5% for the ITOSCs with a MoO*_x_*/Al_2_O_3_:ZnO nanolaminate/PEIE ICL.

As an alternative to PEDOT:PSS, solution‐processed polyoxometalate (POM) has also been utilized as a high WF HTL to combine with ZnO ETL as an ICL.^37^ Using a bilayer P_2_Mo‐POM 18/ZnO ICL to create an intermediate recombination contact, an efficient (9.9% PCE) ITOSC was fabricated with a front cell of P3HT:ICBA and a back cell of PTB7:PC_71_BM. Additionally, all‐solution‐processed GO:SWCNTs/ZnO and graphene/TiO*_x_* were designed for ITOSCs.^275^, ^335^ Although the corresponding device performance is still not comparable to that of ITOSCs with regular MoO*_x_*‐ or PEDOT:PSS‐based ICLs, the carbon‐based materials may still hold a good potential as a general platform to assemble h‐e‐type ICLs for developing diverse type of tandem devices beyond ITOSCs.

## 5 Concluding Remarks and Outlook

In summary, as key components in OSCs, cathode, anode and interconnecting interface layer materials play an important role in determining the performance as well as device stability of OSCs. A great number of electron or hole transporting materials including inorganic metal oxides, organic polymers/small‐molecules, carbon‐based materials, metal salts/complexes, organic‐inorganic hybrids/composites, and other alternatives are presented as effective interfacial layers for high performance OSCs. Interface layers with different structures and morphologies will have varied properties such as energy bandgap, optical transmittance, carrier mobility, conductivity, work function, and surface energy, etc., thereby affecting the performance of OSCs based on them. Through the choice of materials and processing methods, various interface layers are made with desired optoelectronic properties, tunable energy levels and WFs, improved interfacial compatibility, good chemical/physical stability, etc, all of which in combination with the availability of high performance active layer materials have contributed to the benchmark PCEs over 10% for both conventional and inverted OSCs.

Although significant progresses in PCEs of OSCs have been made in the past decade, long‐term stability of these OSCs, a major challenge in the field, is less explored more or less. Therefore, future works should be focused on developing new interfacial materials and active layer materials towards both highly efficient and long‐term stable OSCs, while the degradation mechanism related with interface layers is particularly needed to be made clear. We also know that solution processing is one of most attractive merits of OSCs. Indeed, interfacial materials with large‐area solution processability are crucial for the production of cost‐effective OSCs. Thus the development of all‐solution‐processed electron/hole‐transporting materials for effective CILs, AILs, ICLs in single‐junction or tandem OSCs is needed. Meanwhile, high performance interfacial materials with good universality for different OPV systems are rare. Moreover, the thicknesses of reported interfacial layers are mostly less than 50 nm up to date, which is not beneficial for the roll‐to‐roll production. Therefore, the achievement of thick and efficient CILs, AILs and ICLs with thickness‐insensitive performance is specifically needed for large‐area and low‐cost OSCs in practical applications.

For the future development of OSCs, the careful choice of interfacial materials via a better understanding their roles will be crucial to further improve device efficiency and stability. At the same time, the intensive exploration on understanding the interfacial dynamics and the influencing mechanisms of interfacial layers on the device performance are also very helpful to design next generation high‐performance OSCs. With the rapid development of material innovation and device engineering, high efficiency OPV devices and modules have a promising commercialization future. Recent advances in the interfacial materials have unlocked a wide range of chances for their applications in other photovoltaic systems, such as hybrid solar cells, kesterite/perovskite solar cells, and quantum dot solar cells, etc. It is not surprising to witness further advances in OSCs and other promising photovoltaic devices based on the emerging interfacial materials.

For the tandem OSC application, the choice of active layer materials with complementary absorption bands is very important for boosting PCEs of OSCs to a new high level, in addition to the interfacial materials discussed in this review. So far, similar absorption bands of most active layer materials have restrained the light harvesting range of multi‐junction OSCs, thereby leading to the barely increased PCEs in comparison with the single‐junction devices. Therefore, novel high performance donor/acceptor active layer materials with aborption extended to the near‐IR region are of great importance in order to enrich the choice of materials used for multi‐junction OSCs.
